# Syntactic Computation in the Human Brain: The Degree of Merger as a Key Factor

**DOI:** 10.1371/journal.pone.0056230

**Published:** 2013-02-20

**Authors:** Shinri Ohta, Naoki Fukui, Kuniyoshi L. Sakai

**Affiliations:** 1 Department of Life Sciences, Graduate School of Arts and Sciences, The University of Tokyo, Komaba, Meguro-ku, Tokyo, Japan; 2 Japan Society for the Promotion of Science, Ichiban-cho, Chiyoda-ku, Tokyo, Japan; 3 Department of Linguistics, Sophia University, Kioi-cho, Chiyoda-ku, Tokyo, Japan; 4 CREST, Japan Science and Technology Agency, Goban-cho, Chiyoda-ku, Tokyo, Japan; 5 Department of Basic Science, Graduate School of Arts and Sciences, The University of Tokyo, Komaba, Meguro-ku, Tokyo, Japan; SUNY Downstate MC, United States of America

## Abstract

Our goal of this study is to characterize the functions of language areas in most precise terms. Previous neuroimaging studies have reported that more complex sentences elicit larger activations in the left inferior frontal gyrus (L. F3op/F3t), although the most critical factor still remains to be identified. We hypothesize that pseudowords with grammatical particles and morphosyntactic information alone impose a construction of syntactic structures, just like normal sentences, and that “the Degree of Merger” (DoM) in recursively merged sentences parametrically modulates neural activations. Using jabberwocky sentences with distinct constructions, we fitted various parametric models of syntactic, other linguistic, and nonlinguistic factors to activations measured with functional magnetic resonance imaging. We demonstrated that the models of DoM and “DoM+number of Search (searching syntactic features)” were the best to explain activations in the L. F3op/F3t and supramarginal gyrus (L. SMG), respectively. We further introduced letter strings, which had neither lexical associations nor grammatical particles, but retained both matching orders and symbol orders of sentences. By directly contrasting jabberwocky sentences with letter strings, localized activations in L. F3op/F3t and L. SMG were indeed independent of matching orders and symbol orders. Moreover, by using dynamic causal modeling, we found that the model with a inhibitory modulatory effect for the bottom-up connectivity from L. SMG to L. F3op/F3t was the best one. For this best model, the top-down connection from L. F3op/F3t to L. SMG was significantly positive. By using diffusion-tensor imaging, we confirmed that the left dorsal pathway of the superior longitudinal and arcuate fasciculi consistently connected these regions. Lastly, we established that nonlinguistic order-related and error-related factors significantly activated the right (R.) lateral premotor cortex and R. F3op/F3t, respectively. These results indicate that the identified network of L. F3op/F3t and L. SMG subserves the calculation of DoM in recursively merged sentences.

## Introduction

It is widely accepted that in human language, a sentence can be expressed by a unique tree structure with recursive branches [1], [2]. Moreover, any sentence can be recursively combined within another sentence, as in e.g., “*I think that John believes that Mary assumes that…*”, and there is in principle no upper bound for the length of sentences; this property is the so-called *discrete infinity* made possible by the computational power, or engine, of the human language faculty. One possible way to elucidate the neural basis of such computational properties is to examine how the brain responds to the modulation of specified syntactic factors. An early attempt with functional magnetic resonance imaging (fMRI) has reported that activations in the language areas were modulated by noncanonical/canonical word orders and the presence/absence of lexical contents [3], in which multiple factors, including memory-related and semantic factors, could account for these activations. Therefore, we should not be content with such a general cognitive phenomenon as so-called “syntactic complexity” or “syntactic working memory” that could involve both linguistic and nonlinguistic factors. We should instead identify which minimal factor *sufficiently* explains any activation changes obtained. In addition, the size of linguistic constituents may also modulate cortical activations. A recent fMRI study has reported that the left frontal activations increased with the number of words or terminal nodes (symbols) in a phrase [4], but, as rightly pointed out by the authors, the precise phrase structures remained to be taken into account. Here we focus on different sentence constructions, and try to identify *minimal* syntactic factors associated with phrase structures, which parametrically modulate cortical responses measured with event-related fMRI.

**Figure 1:**
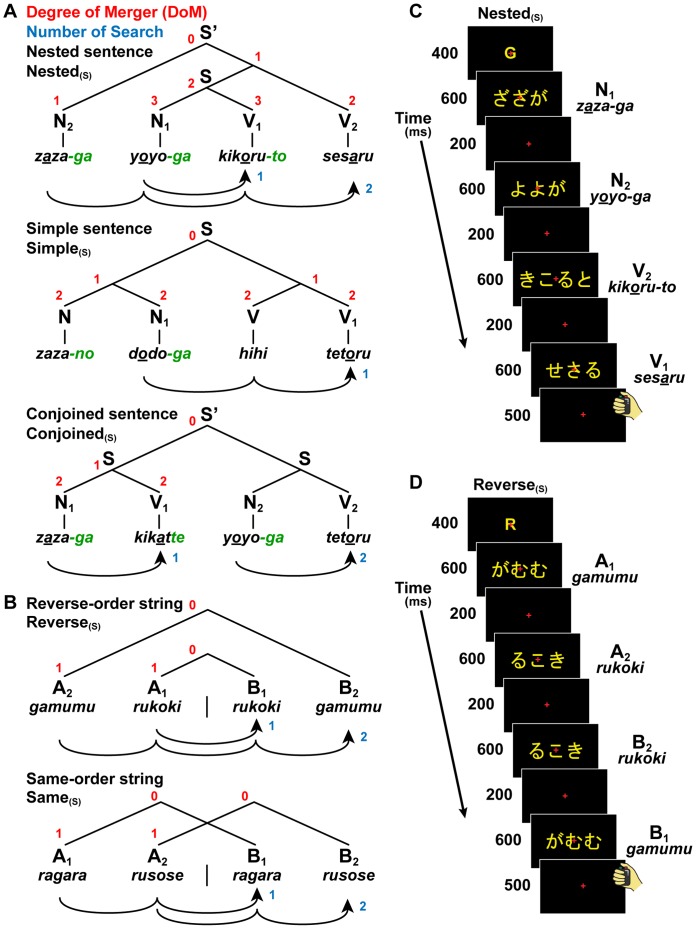
A paradigm for testing jabberwocky sentences and letter strings. Examples of short [(S) as a subscript] matching stimuli are shown here with the Romanization system, but actual stimuli were presented in hiragana without hyphen (see C and D). (A) Three sentence conditions with short stimuli: Nested_(S)_, Simple_(S)_, and Conjoined_(S)_. Based on contemporary linguistics [2], each diagram represents a unique tree structure of each sentence (S and S’) constructed from Ns and Vs. For the Nested_(S)_, a sentence (S) at the lowest hierarchical level was nested into an entire sentence (S’), similar to “*Taro-ga Hanako-ga utau-to omou*” (“*Taro thinks that Hanako sings*”). For the Simple_(S)_, a simple sentence was constructed by adding the same number of left/right branches to both Ns and Vs, similar to “*Taro-no ani-ga tabe hajimeru*” (“*Taro’s brother starts eating*”). For the Conjoined_(S)_, an entire sentence (S’) was constructed by conjoining two sentences, similar to “*Taro-ga utatte Hanako-ga odoru*” (“*Taro sings, and Hanako dances*”). The digits shown in red and blue denote DoM for each node and “number of Search”, respectively (see Table 1). The curved arrows denote the matching of sequentially presented stimuli. (B) Two string conditions with short stimuli: Reverse_(S)_ and Same_(S)_. Each letter string was formed by jumbling letters of either N or V. (C and D) Examples of stimulus presentation. Here, examples of matching stimuli are shown in hiragana for the Nested_(S)_ and Reverse_(S)_. Between the Nested_(S)_ and Reverse_(S)_, both of the symbol orders (the order of Ns, Vs, As, and Bs) and matching orders (denoted by subscripts) were identical.

Modern linguistics has accumulated mounting evidence that the construction of any grammatical phrases or sentences can be adequately and *minimally* explained by hierarchical syntactic structures with a set of relevant structural relations defined on such structures [5], [6], leading to the postulation of the fundamental linguistic operation of *Merge* (capitalized in linguistics to indicate formal operations), the structure-building operation, which combines two syntactic objects (words or phrases) to form a larger structure [7]. Besides Merge, we have proposed that *Search* (searching syntactic features) applies to a syntactic object already constructed by Merge, and that Search assigns relevant features to the syntactic object [8]. The total number of Merge and Search applications within an entire sentence are here simply denoted as “number of Merge” and “number of Search”, respectively. To properly measure the depth of a tree structure with a formal property of Merge and *iterativity* (recursiveness) [9], we hypothesize that “the Degree of Merger (DoM)” is a key computational concept, which can be defined as the *maximum depth* of merged subtrees (i.e., Mergers) within an entire sentence. Moreover, DoM can quantify and compare various syntactic phenomena, such as self-embedding, scrambling, wh-movement, etc. Furthermore, when Search applies to each syntactic object within hierarchical structures, the calculation of DoM plays a critical role. Indeed, from a nested sentence “[[*The boy*_2_ [*we*_3_ *like*_3_]_2_]_1_ *sings*_1_]_0″_ (subscripts denote DoM for each node, see Figure 1A), two sentences “[*The boy* … ]_1_ *sings*_1_” and “*we*_3_ *like*_3_” are obtained, where relevant features (numbers and persons here) are searched and checked between the nodes with identical DoM. Because such analyses of hierarchical structures would produce specific loads in syntactic computation, we expect that DoM and associated “number of Search” modulate neural activations. Merge would be theoretically “costless” [10], [11], and thus “number of Merge” itself may not affect activations, which can be easily expected for *flat* structures (see Figure 1B).

**Figure 2:**
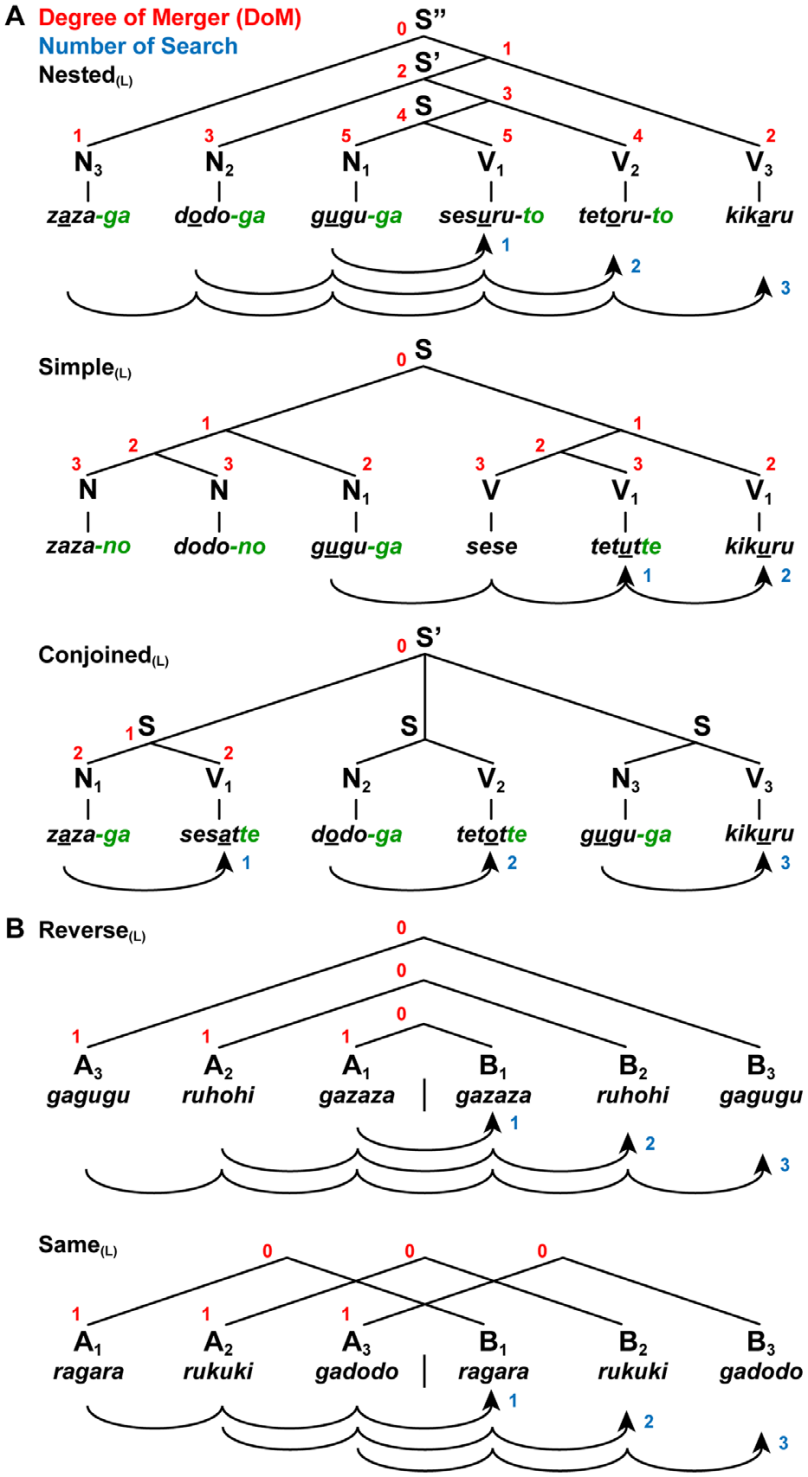
Examples of long matching stimuli. (A) Three sentence conditions with long [(L) as a subscript] stimuli: Nested_(L)_, Simple_(L)_, and Conjoined_(L)_. (B) Two string conditions with long stimuli: Reverse_(L)_ and Same_(L)_. See Appendix S2 for further explanation.

In the present study, jabberwocky sentences that lacked lexical associations were prepared. Each sentence consisted of pseudonoun phrases (Ns) and pseudoverb phrases (Vs). We hypothesize that pseudowords with grammatical particles and morphosyntactic information alone impose a construction of syntactic structures, just like normal sentences (see **Materials and Methods**, Stimuli). Based on the nested (self-embedded), left/right-branching, and multiple-branching constructions (see Appendix S1), we introduced three basic types of sentence constructions: nested sentence (Nested), simple sentence (Simple), and conjoined sentence (Conjoined) (Figure 1A). When constructing syntactic structures like the ones shown in Figure 1A, the correspondence of each subject-verb pair is most crucial. To test that participants actually paid attention to this correspondence, we used a matching task, such that the vowel of a subject (N_i_ as a sample stimulus) was matched with the last vowel of the corresponding verb root (V_i_ as a comparison stimulus) (e.g., “*zaza-ga sesaru*”, underlined vowels within pseudowords). These features of vowels were only *experimentally* introduced, and this matching involved a factor of encoding (i.e., memorization of features necessary for matching). Because Vs lacked grammatical (agreement) features (e.g., number, person, gender, etc.), as in the Japanese verbs, this property of matching did not mimic agreement itself, but involved a formal association between sample and comparison stimuli. It follows that the same syntactic structures were constructed from matching and nonmatching stimuli (Tables S1 and S2), which were both well-formed, i.e., *grammatical*, in Japanese. Matching strategy (counting, for example, first and fourth stimuli for matching) was useful in solving the task, but performing the task was *not* prerequisite for constructing syntactic structures. Our matching task is different from classification tasks for symbol orders (e.g., AABB vs. ABAB, where A and B are symbols representing certain sets of stimuli), which can be solved by counting the number of each set, A or B. We further examined whether cortical activations were modulated by the length of sentences: short (S as a subscript, four-phrase) and long (L as a subscript, six-phrase) sentences (Figure 2A).

We tested various parametric models of syntactic, other linguistic, and nonlinguistic factors (Table 1; see Appendix S1 for operational definitions), some of which were based on structure-based models (Figures S1, S2, S3). Given these factors with a limited number of experimental conditions, we wanted to narrow down the models as much as possible by adopting effective contrasts. For both short and long sentences, the estimates of “number of Merge”, as well as those of “number of case markers (*-ga*/-*no*)” and “depth of postponed symbols”, were identical among the three sentence conditions. By taking one of sentence conditions as a reference, these three factors could be eliminated from the analyses. Moreover, a reference condition should be chosen separately for each of short and long sentences, as we tested the short and long stimuli on separate days. The Conjoined condition was actually *simplest* among the three sentence conditions and thus served as an appropriate reference, because the Conjoined condition had same or less estimates than those under the Nested and Simple conditions for all factors except the numbers of Search and encoding. For brevity, a contrast with the Conjoined condition as a reference is denoted with a prime mark; e.g., [Nested_(S)_ – Conjoined_(S)_] and [Nested_(L)_ – Conjoined_(L)_] abbreviated as Nested’_(S)_ and Nested’_(L)_, respectively.

**Table 1:** Estimates of various factors to account for activations under the sentence conditions.

Syntacticfactors	Factor	Nested_(L)_	Nested_(S)_	Simple_(L)_	Simple_(S)_	Conjoined_(L)_	Conjoined_(S)_
	Degree of Merger (DoM)	5	3	3	2	2	2
	No. of Search	3	2	2	1	3	2
	No. of Merge	5	3	5	3	5	3
		**Nested’_(L)_**	**Nested’_(S)_**	**Simple’_(L)_**	**Simple’_(S)_**		
	DoM	3	1	1	0		
	DoM + No. of Search	3	1	0	–1		
	No. of Search	0	0	–1	–1		
	No. of Merge	0	0	0	0		
**Other linguistic** **factors**	**Factor**	**Nested_(L)_**	**Nested_(S)_**	**Simple_(L)_**	**Simple_(S)_**	**Conjoined_(L)_**	**Conjoined_(S)_**
	No. of case markers (*-ga*/-*no*)	3	2	3	2	3	2
	No. of tense markers (*-ru*/-*ta*)	3	2	1	1	1	1
	Degree of nesting	2	1	1	1	1	1
	Degree of self-embedding	2	1	1	0	1	0
	No. of nodes	11	7	11	7	10	7
	Depth of postponed symbols	3	2	3	2	3	2
	Integration costs	5	3	3	2	1	1
	Storage costs	3	2	2	2	1	1
	Syntactic interference	2	1	0	0	0	0
	Positional similarity	3	2	2	0	0	0
		**Nested’_(L)_**	**Nested’_(S)_**	**Simple’_(L)_**	**Simple’_(S)_**		
	No. of case markers (*-ga*/-*no*)	0	0	0	0		
	No. of tense markers (*-ru*/-*ta*)	2	1	0	0		
	Degree of nesting	1	0	0	0		
	Degree of self-embedding	1	1	0	0		
	No. of nodes	1	0	1	0		
	Depth of postponed symbols	0	0	0	0		
	Integration costs	4	2	2	1		
	Storage costs	2	1	1	1		
	Syntactic interference	2	1	0	0		
	Positional similarity	3	2	2	0		
**Nonlinguistic** **factors**	**Factor**	**Nested_(L)_**	**Nested_(S)_**	**Simple_(L)_**	**Simple_(S)_**	**Conjoined_(L)_**	**Conjoined_(S)_**
	Memory span	4	2	2	1	0	0
	Counting	2	1	2	1	0	0
	No. of encoding	6	4	3	2	6	4
		**Nested’_(L)_**	**Nested’_(S)_**	**Simple’_(L)_**	**Simple’_(S)_**		
	Memory span	4	2	2	1		
	Counting	2	1	2	1		
	No. of encoding	0	0	–3	–2		
	Memory span + counting	6	3	4	2		
	Memory span + No. of encoding	4	2	–1	–1		

We define the estimate of a factor as the largest value that the factor can variably take within an entire sentence. For each factor, its unit load should be invariable among all sentence conditions, making an independent subtraction between estimates of the *same* factor possible. Separately for long and short sentences, estimates under the Conjoined condition as a reference were subtracted from those under the other Nested and Simple conditions (e.g., the [Nested_(L)_ – Conjoined_(L)_] contrast abbreviated as Nested’_(L)_; e.g., DoM for Nested’_(L)_, 5–2 = 3). Excluding “number of Merge”, the estimates of which were null for all four contrasts, we regarded “DoM+number of Search” (i.e., adding the estimates of two factors) as an additional factor. Among the nonlinguistic factors, “memory span+counting” and “memory span+number of encoding” were regarded as additional factors, because they were temporal order-related and memory-related factors, respectively.

We further introduced letter strings, which had neither lexical associations nor grammatical particles, but retained both matching orders and symbol orders of sentences. There were two string conditions: reverse-order string (Reverse) and same-order string (Same) (Figures 1B, 2B, and Table 2). Like the sentence conditions, we used the same matching task under these string conditions, such that the first half of a string (A_i_ as a sample stimulus) was matched with the corresponding second half (B_i_ as a comparison stimulus) in the reverse or same order. These string conditions also controlled any involvement of matching strategy stated above. Between the Nested (N_2_ N_1_ V_1_ V_2_ or N_3_ N_2_ N_1_ V_1_ V_2_ V_3_, where each subscript denotes a matching order) and Reverse (A_2_ A_1_ B_1_ B_2_ or A_3_ A_2_ A_1_ B_1_ B_2_ B_3_) conditions, the curved arrows shown in Figures 1 and 2 represent the *same* matching order of sequentially presented stimuli (e.g., for N_2_ N_1_ V_1_ V_2_, the inner symbol pair of N and V is matched first, and then the outer symbol pair is matched). The symbol order was also identical among the Nested, Simple, Reverse, and Same conditions in the form of N*^n^* V*^n^* or A*^n^* B*^n^*. To control both matching orders and symbol orders, we directly compared the Nested with the Reverse, using the Simple and Same conditions as respective references (Table 2), i.e., (Nested – Simple)>(Reverse – Same). For brevity, the contrasts of [Nested – Simple] and [Reverse – Same] are denoted with a double prime mark, i.e., Nested” and Reverse”, respectively. Our goal with such thorough controls was to demonstrate that *purely* syntactic factors of DoM and “number of Search” actually modulate neural activations.

**Table 2:** Estimates of nonlinguistic and syntactic factors to account for activations.

Nonlinguistic factors	Factor	Nested_(L)_	Nested_(S)_	Simple_(L)_	Simple_(S)_	Reverse_(L)_	Reverse_(S)_	Same_(L)_	Same_(S)_
	Memory span	4	2	2	1	4	2	2	1
	Counting	2	1	2	1	2	1	2	1
	No. of encoding	6	4	3	2	6	4	6	4
		**Nested**		**Simple**		**Reverse**		**Same**	
	Memory span	6		3		6		3	
	Counting	3		3		3		3	
	No. of encoding	10		5		10		10	
		**Nested”**				**Reverse”**			
	Memory span	3				3			
	Counting	0				0			
	No. of encoding	5				0			
**Syntactic factors**	**Factor**	**Nested”**				**Reverse”**			
	DoM	3				0			
	DoM+No. of Search	5				0			

For Nested, Simple, Reverse, and Same, the estimates for short and long stimuli were added together, because each factor’s unit load would be invariable between short and long stimuli under each of the sentence and string conditions. Because the matching orders or symbol orders were identical between the Nested and Reverse conditions, the unit load of memory span or counting was invariable between the Nested and Reverse conditions, which was also invariable between the Reverse and Same conditions, thus invariable among the Nested, Simple, Reverse, and Same conditions. For brevity, the contrasts of [Nested – Simple] and [Reverse – Same] are denoted with a double prime mark, i.e., Nested” and Reverse”, respectively. Note that the estimates of memory span in Nested” and Reverse” also became identical, and that the Reverse” contrast makes the listed estimates null, except memory span. The last two syntactic factors, whose models were best in Table 5, consistently accounted for the results of Figure 4F. All estimates of the other factors unlisted here were null in Reverse”, which cannot account for the results of Figure 5C and 5D.

It has been reported that more complex sentences elicit larger activations in the pars opercularis and pars triangularis of the left inferior frontal gyrus (L. F3op/F3t) [12]–[19], suggesting that L. F3op/F3t is critical for syntactic processing as a grammar center [20]. On the other hand, the left angular and supramarginal gyri (L. AG/SMG) have been suggested for vocabulary knowledge or lexical processing [21], [22]. To examine the functional specialization of any regions, including L. F3op/F3t and L. AG/SMG, in an unbiased manner, we adopted whole-brain analyses [23]. We also performed effective connectivity analyses by using dynamic causal modeling (DCM) [24] to examine the functional integration of identified regions. To provide empirical backup for the connection derived from DCM, we checked the anatomical plausibility of the network with diffusion-tensor imaging (DTI). According to recent DTI studies, there have been controversial issues as regards the functional roles of two different pathways for syntax, semantics, and phonology: dorsal tracts of the superior longitudinal and arcuate fasciculi (SLF/AF), as well as ventral tracts of the middle longitudinal fasciculus (MdLF) and extreme capsule (EmC); both pathways connect the inferior frontal and superior/middle temporal areas [25]–[28]. Our present study would elucidate the most crucial network and pathway for syntactic computation.

## Materials and Methods

### Participants

Eighteen native Japanese speakers (all males, aged 19–25 years), who had not majored in linguistics, participated in an fMRI experiment. Additional 15 participants (14 males, aged 19–40 years) were tested in a DTI experiment. All participants in the fMRI and DTI experiments were healthy and right-handed (laterality quotients: 11–100), according to the Edinburgh inventory [29]. Prior to participation in the study, written informed consent was obtained from each participant after the nature and possible consequences of the studies were explained. Approval for the experiments was obtained from the institutional review board of the University of Tokyo, Komaba.

### Stimuli

Each visual stimulus consisted of two to five yellow letters in hiragana (Figure 1C and 1D). The stimuli were visually presented against a dark background through an eyeglass-like MRI-compatible display (resolution, 800×600; VisuaStim XGA; Resonance Technology Inc., Northridge, CA). The visual stimuli were always presented at the center of the monitor. At the initiation of every trial of the Nested, Simple, and Conjoined, the cue “G” (for grammar conditions with all grammatical sentences) was shown for 400 ms. The cue “R” (for reverse orders) was shown for the Reverse, and “M” (for memorizing orders) for the Same. Four (short) or six (long) stimuli were each sequentially presented to the participants for 600 ms, with an interstimulus interval of 200 ms, leading to 4.5 s and 6 s trials for the short and long stimuli, respectively. For fixation, a red cross was always displayed at the center of the monitor. During fMRI experiments, stimulus presentation, as well as acquisition of responses and reaction times (RTs), was controlled using the LabVIEW software and interface (National Instruments, Austin, TX).

Under the sentence conditions, jabberwocky sentences consisting of pseudonoun phrases and pseudoverb phrases alone were presented in a phrase-by-phrase manner to the participants. We made six pseudonouns by repeating the same syllables with voiced consonants and any one of/a/,/u/, or/o/: *rara*, *zaza*, *mumu*, *gugu*, *yoyo*, and *dodo*. We also made four pseudoverb roots by repeating the same syllables with voiceless consonants and either/i/or/e/: *kiki*, *hihi*, *sese*, and *tete*. The transitions between consecutive phrases or sentences were thoroughly randomized. Nonmatching stimuli included at least one odd vowel of V_i_ as a matching error (Tables S1 and S2). All matching and nonmatching stimuli were phonotactically legal, but lacked lexical associations in Japanese. There were 10 conditions (Figures 1 and 2); we prepared a set of 36 sentences for each of sentence conditions, and a set of 36 letter strings for each of string conditions. Each set consisted of 18 matching and 18 nonmatching stimuli. See Appendix S2 for detailed information about the stimuli.

We used only three kinds of grammatical particles, which represent *canonical* (i.e., in a prototypical use) case markings and syntactic information in Japanese: *-ga*, a nominative case marker; *-no*, a genitive case marker; and *-to*, a complementizer. In all jabberwocky sentences, the distinction between Ns and Vs was clear without memorizing pseudowords, because Ns, but not Vs, ended with either *-ga* or *-no*; only nouns and pronouns precede case markers in Japanese (e.g., “*momo-ga minoru*” and “*momo-no iro*”: “*the peach ripens*” and “*the peach’s color*”; real phrases will be translated hereafter). Moreover, Vs took a nonpast-tense form (*-ru*), past-tense form (*-ta*), or gerundive form (*-te*), following morphosyntactic and phonological features of Japanese verbs [30]; Vs ended with *-to* and *-te* introduced *that*-clauses and *and*-conjunctives, respectively (see examples in Figure 1 legend). Including the first verb of a compound verb in an adverbial form (e.g., “*hihi*” and “*sese*”), all Ns and Vs with *-ga*, *-no*, *-to*, and *-te* endings (green letters in Figures 1A and 2A) were associated with Merge applications to connect multiple nouns/verbs or sentences, amounting to “number of Merge”.

Under the string conditions, stimuli were presented in the reverse order for the Reverse, whereas they were in the same order for the Same, as regards the first and second halves of a string (Figures 1B and 2B). Each letter string was formed by jumbling letters of either N or V, which had no lexical associations. For the Reverse and Same, there was actually no path connecting the nonterminal nodes of symbol pairs (e.g., A_1_ B_1_ and A_2_ B_2_), as there was *no* Merge application to connect the multiple pairs. The letter strings lacked *-ga*, *-no*, *-to*, or *-te* endings, and their flat constructions were determined by the cue of “R” or “M” alone. We estimated the syntactic factors for the letter strings, but all estimates of these factors were null in Reverse” (see Table 2).

### Task

For each trial of a matching task under the sentence conditions or string conditions, the participants judged whether or not all pairs of the sample stimulus (N or A) and comparison stimulus (V or B) were matched, and responded by pressing one of two buttons (right for matching, and left for nonmatching) after the last stimulus appeared (Figure 1C and 1D). The accuracy and RTs were collected until 500 ms after the last stimulus disappeared. No feedback on each trial’s performance was given to any participant. See Appendix S3 for task instructions and training procedures.

For the Nested, an entire sentence was constructed by nesting sentences in the form of [N_2_[N_1_ V_1_]V_2_] or [N_3_[N_2_[N_1_ V_1_]V_2_]V_3_], where [N_i_ V_i_] represents a subject-verb pair of a sentence (Figures 1A and 2A). In head-last languages, the key element (the “head”) that determines the properties of a phrase is placed at the end of the phrase. Because Japanese is a head-last, and hence an SOV (verb-final) language, a main verb is placed after a subordinate clause. Therefore, Japanese sentences naturally yield nested structures of N*^n^* V*^n^* without having to employ, as in English, object-relative clauses (e.g., “*The boy who_i_ we like t_i_ sings*”), which require “movement” of an object (i.e., with more Merge applications) leaving behind a “trace” (*t_i_*). For the Simple, a simple sentence was constructed by adding the same number of left/right branches to both Ns and Vs. The last noun (i.e., head) in the branches of Ns made a subject-verb pair with the last verb (i.e., head) of a compound verb. Each simple sentence thus took the form of [(NN_1_) (VV_1_)], etc. For the Conjoined, an entire sentence was constructed by conjoining sentences in the form of [N_1_ V_1_][N_2_ V_2_] or [N_1_ V_1_][N_2_ V_2_][N_3_ V_3_].

In a single run of 60 trials for the short stimuli, there were 10 trials each for the sentence conditions (the Nested_(S)_, Simple_(S)_, and Conjoined_(S)_), and 15 trials each for the string conditions (the Reverse_(S)_ and Same_(S)_). Each trial was alternately a sentence condition and a string condition. If the sentence and string sequences were separated, the order of the Nested, Simple, and Conjoined was pseudo-randomized without repetition, and the order of the Reverse and Same was counterbalanced as Same-Reverse-Reverse-Same-… or Reverse-Same-Same-Reverse-… In a single run of 50 trials for the long stimuli, there were 10 trials each for the sentence conditions (the Nested_(L)_, Simple_(L)_, and Conjoined_(L)_) and the string conditions (the Reverse_(L)_ and Same_(L)_), in the order of string-sentence-string-sentence-sentence-string-… With a maximum of nine runs, the same sentence stimulus appeared no more than three times for each participant.

### MRI Data Acquisition

Depending on the time of experiments, the fMRI scans were conducted on a 1.5 T scanner (Stratis II, Premium; Hitachi Medical Corporation, Tokyo, Japan) with a bird-cage head coil, and the DTI scans were conducted on a 3.0 T scanner (Signa HDxt; GE Healthcare, Milwaukee, WI) with an 8-channel phased-array head coil. For the fMRI, we scanned 26 axial slices that were 3-mm thick with a 1-mm gap, covering from *z* = –40 to 63 mm from the anterior to posterior commissure (AC-PC) line, with a gradient-echo echo-planar imaging (EPI) sequence [repetition time (TR) = 3 s, echo time (TE) = 51 ms, flip angle (FA) = 90°, field of view (FOV) = 192×192 mm^2^, resolution = 3×3 mm^2^]. In a single scanning run, we obtained 92 volumes for the short stimuli and 101 volumes for the long stimuli following three dummy images, which allowed for the rise of the MR signals. For each participant, five to nine runs for each of the short and long stimuli were tested, and four to nine runs without head movement were used for analyses. After completion of the fMRI session, high-resolution T1-weighted images of the whole brain (145 axial slices, 1×1×1 mm^3^) were acquired from all participants with a radio frequency spoiled steady-state acquisition with a rewound gradient echo sequence (TR = 30 ms, TE = 8 ms, FA = 60°, FOV = 256×256 mm^2^).

For the DTI, we scanned 50 axial slices that were 3-mm thick without gap, covering from *z* = –60 to 90 mm from the AC-PC line, with a diffusion-weighted spin-echo EPI sequence (b-value = 1,000 s/mm^2^, TR = 15 s, TE = 87 ms, FOV = 256×256 mm^2^, resolution = 2×2 mm^2^, number of excitations = 2). A single image without diffusion-weighting (b0) was initially acquired, and then diffusion-weighting was isotropically distributed along 60 diffusion-encoding gradient directions. After completion of the DTI sessions, high-resolution T1-weighted images of the whole brain (192 axial slices, 1×1×1 mm^3^) were acquired from all participants with a fast spoiled gradient recalled acquisition in the steady state sequence (TR = 10 ms, TE = 4 ms, FA = 25°, FOV = 256×256 mm^2^). See Appendix S4 for MRI data analyses.

## Results

### Condition and Length Effects on the Accuracy/RTs

**Figure 3:**
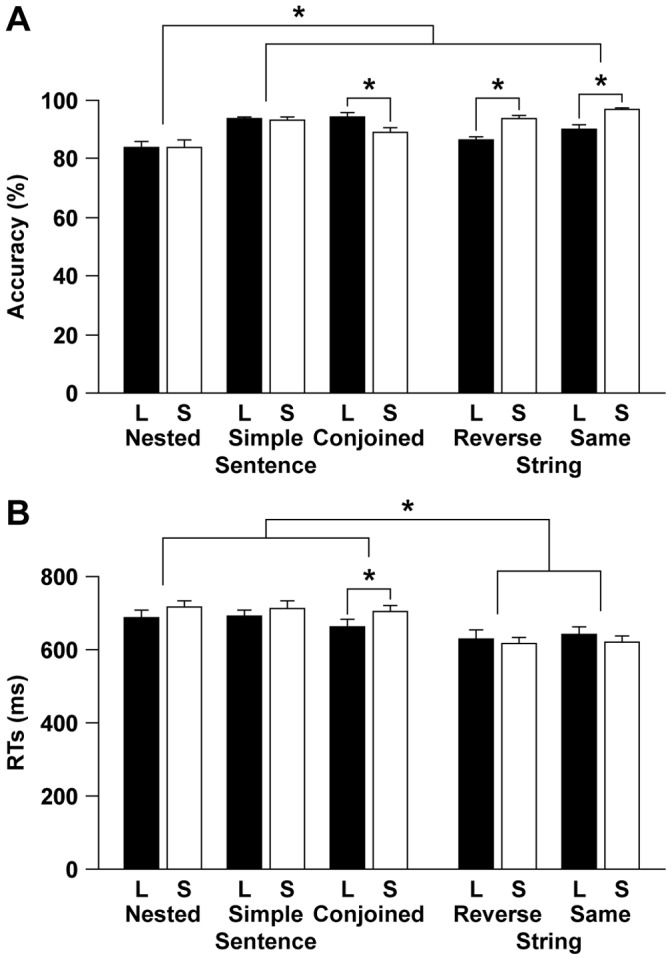
Condition and length effects on the accuracy/RTs. (A) The accuracy (mean ± SEM) for long (L) and short (S) stimuli, denoted by filled and open bars, respectively. Asterisks indicate the significance level at corrected *P*<0.05 (paired *t*-tests). (B) RTs from the onset of the last stimulus.

The accuracy data, as well as RTs measured from the onset of the last stimulus, are shown in Figure 3. The high accuracy under both sentence and string conditions indicated the participants’ reliable and consistent judgments on the matching task. A two-way repeated-measures analysis of variance (rANOVA) with the condition [Nested, Simple, Conjoined, Reverse, Simple]×length [Long, Short] for the accuracy showed a significant main effect of condition [*F*(4, 68) = 15, *P*<0.0001] and an interaction of condition by length [*F*(4, 68) = 12, *P*<0.0001], but a main effect of length was not significant [*F*(4, 68) = 3.8, *P* = 0.07]. The RTs also showed a significant main effect of condition [*F*(4, 68) = 43, *P*<0.0001] and an interaction of condition by length [*F*(4, 68) = 13, *P*<0.0001], but a main effect of length was not significant [*F*(4, 68) = 1.1, *P* = 0.30]. Post-hoc paired *t*-tests among all conditions (significance level at *α* = 0.005, Bonferroni corrected) showed that the accuracy for the Nested was significantly lower than that under the other conditions including the Reverse (*P*<0.0001). This result indicates that the Nested was the most demanding condition, which cannot be explained by the *nonlinguistic* factors we examined (cf. the same estimates for the Nested and Reverse in Table 2, as well as its notes). On the other hand, post-hoc paired *t*-tests showed that the RTs under each sentence condition were significantly longer than those under each string condition (*P*<0.0001). This difficulty was not in the task itself, but in vowel extraction; the sentence conditions, but not the string conditions, involved vowel extraction from the second syllable of V_i_ presented in hiragana, especially for the last V_i_ that were directly linked with RTs (Figure 1C and 1D). The load of vowel extraction would become also larger for the *short* stimuli, as we tested the short and long stimuli on separate days in the order short, then long. Indeed, the accuracy for the Conjoined_(S)_ was significantly lower than that for the Conjoined_(L)_ [*t*(17) = 3.1, *P* = 0.006] (significance level at *α* = 0.01, Bonferroni corrected), and the RTs for the Conjoined_(S)_ were significantly longer than those for the Conjoined_(L)_ [*t*(17) = 2.8, *P* = 0.01], probably reflecting associated effects for novices. For the Conjoined, length effects were apparently absent, and the estimates of both memory span and counting, which were associated with length effects, were indeed null for the Conjoined alone (Table 1). In the present study, we mainly analyzed activations that would show length effects (i.e., Long>Short), excluding the involvement of vowel extraction or effects for novices. Moreover, we used the Conjoined condition, which showed such effects most strongly, as a reference for both Nested and Simple conditions. Therefore, we can safely conclude that any elicited effects did not directly relate to the task.

Under the string conditions, the accuracy for the long stimuli was significantly lower than that for the short stimuli (*P*<0.001), indicating length effects. For the Nested and Simple conditions, in contrast, the effects for novices and length would have been cancelled out, as neither the accuracy nor RTs differed significantly between the short and long stimuli (*P*>0.05). Under the string conditions, the accuracy was more sensitive than the RTs.

### Functional Evidence of Syntactic Computation in Language Areas

**Figure 4:**
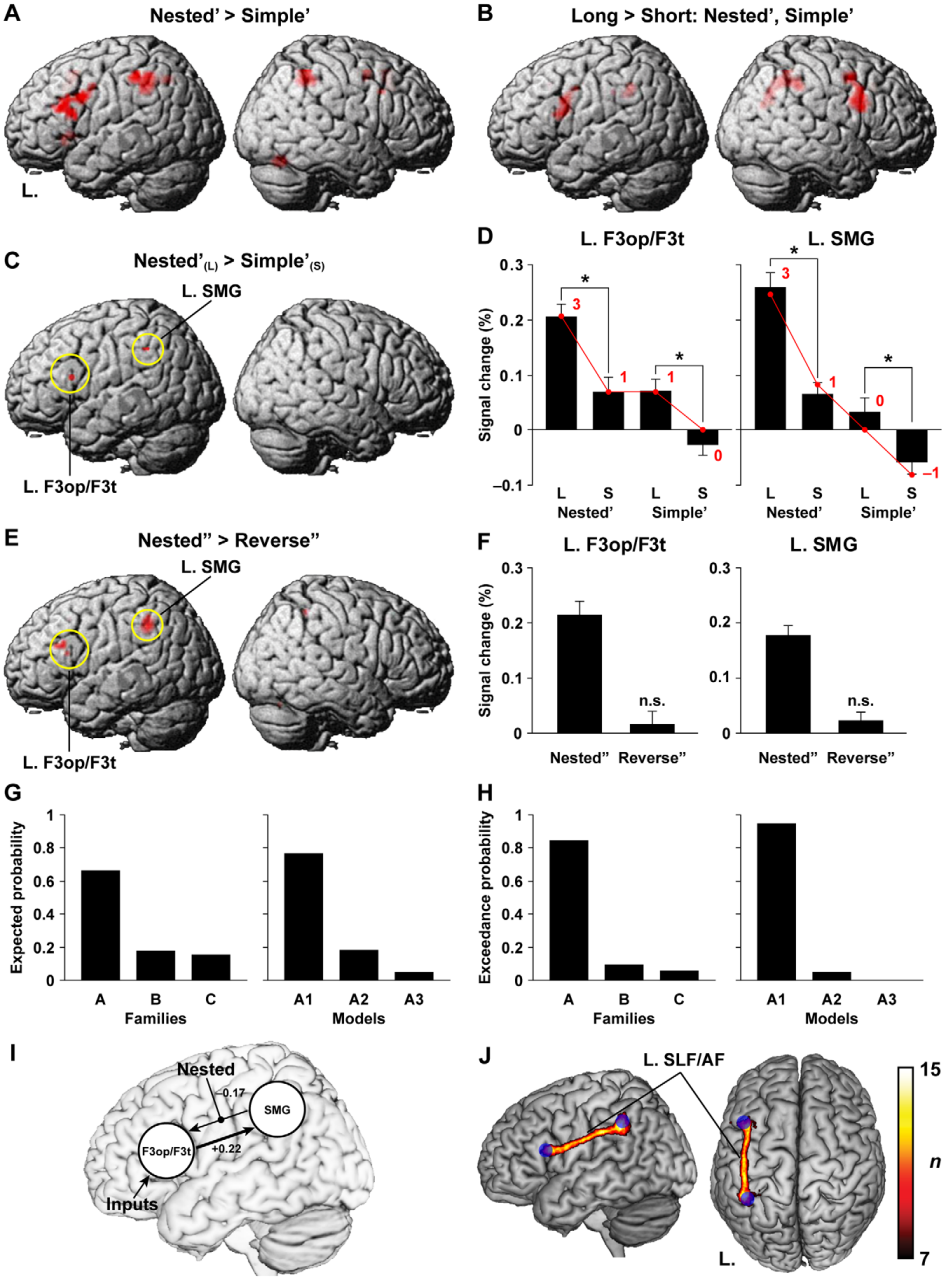
Functional and anatomical evidence of syntactic computation in language areas. For (A) and (B), we used a two-way ANCOVA with condition×length; for (C) and (E), a one-way ANCOVA was used. Activations were projected onto the left (L.) and right lateral surfaces of a standard brain. See Tables 3 and 4 for their stereotactic coordinates. (A) Regions identified by the main effect of condition, i.e., Nested’>Simple’ (Nested’ and Simple’ denote [Nested – Conjoined] and [Simple – Conjoined], respectively). (B) Regions identified by the main effect of length, i.e., Long>Short while combining Nested’ and Simple’. (C) Regions identified by Nested’_(L)_>Simple’_(S)_, which reflected both main effects. (D) Percent signal changes for Nested’ and Simple’, averaged across L. F3op/F3t and L. SMG in (C) (mean ± SEM). Overlaid red dots and lines denote the values fitted with the estimates (digits in red) for the best models: DoM for L. F3op/F3t and “DoM+number of Search” for L. SMG. (E) Regions identified by Nested”>Reverse” (Nested” and Reverse” denote [Nested – Simple] and [Reverse – Same], respectively). (F) Percent signal changes for Nested” and Reverse”, averaged across the L. F3op/F3t and L. SMG in (E). (G–I) The results of DCM, testing effective connectivity between L. F3op/F3t and L. SMG (see Figure S4). Bar graphs show expected probabilities (G) and exceedance probabilities (H) for each modulatory family and for the input models of the winning family *A*. The best model *A1* (I) included a significant intrinsic connection (a thick line). (J) Anatomical connectivity between L. F3op/F3t and L. SMG revealed by DTI. The population probability map is shown on the left lateral and dorsal surfaces of a standard brain with maximum intensity projection. Blue spheres represent seed regions of L. F3op/F3t and L. SMG.

We examined brain activation under the sentence conditions, in particular focusing on selective activations for the most-demanding Nested condition. In a two-way analysis of covariance (ANCOVA) with the condition [Nested’, Simple’]×length [Long, Short], the main effect of condition, i.e., Nested’>Simple’ while combining Long and Short, resulted in left-dominant activation, especially in L. F3op/F3t, left lateral premotor cortex and F3op (L. LPMC/F3op), and L. SMG (Figure 4A and Table 3). Other significantly activated regions were the right (R.) F3op/F3t, R. LPMC, anterior cingulate cortex (ACC), and R. SMG. The main effect of length, i.e., Long>Short while combining Nested’ and Simple’, also showed significant activations in the same regions, while there were more significant voxels in the right hemisphere (Figure 4B). Therefore, length effects alone cannot account for the consistent activation in these regions. An interaction of condition by length did not show any significant activation.

**Table 3:** Regions related to the sentence conditions.

Contrast	Brain region	BA	Side	*x*	*y*	*z*	*Z* Value	Voxels
Main effect of condition, Nested’>Simple’	F3op/F3t	44/45	L	–51	27	24	5.6	109
	LPMC/F3op	6/44	L	–48	9	30	5.4	*
	F3op/F3t	44/45	R	54	15	36	4.9	2
	LPMC	6	R	33	3	51	5.3	12
	Insula	–	L	–30	24	–3	5.7	20
	ACC	6/8/32	M	–3	18	48	6.1	45
	SMG	40	L	–54	–33	48	5.3	101
				–39	–42	39	5.9	*
			R	42	–48	54	5.3	64
	AG/SMG	39/40	L	–30	–60	48	4.9	11
	Cerebellum, lobule VI	–	R	27	–69	–21	5.6	26
Main effect of length, Long>Short:Nested’, Simple’	F3op/F3t	44/45	L	–48	12	18	5.9	63
	LPMC/F3op	6/44	L	–48	3	39	4.7	3
			R	48	6	30	6.0	129
	F3op/F3t	44/45	R	54	12	30	5.8	*
	LPMC	6	R	30	0	48	5.9	*
	ACC	6/8/32	M	0	27	39	4.9	9
	SMG	40	L	–57	–30	36	4.7	1
				–36	–45	39	5.3	26
			R	42	–42	42	5.5	116
	AG/SMG	39/40	R	33	–63	27	5.4	*

Stereotactic coordinates (*x*, *y*, *z*) in the Montreal Neurological Institute (MNI) space (mm) are shown for each activation peak of *Z* values (corrected *P*<0.05). BA, Brodmann’s area; L, left hemisphere; R, right hemisphere; M, medial; F3op/F3t, pars opercularis and pars triangularis of the inferior frontal gyrus; LPMC, lateral premotor cortex; ACC, anterior cingulate cortex; SMG, supramarginal gyrus; AG, angular gyrus. The region with an asterisk is included within the same cluster shown one row above.

To further narrow down candidate regions, we tested Nested’_(L)_>Simple’_(S)_, which reflected both main effects, and found significant activation in L. F3op/F3t and L. SMG (Figure 4C and Table 4). The data used for selecting these regions and those for subsequent analyses were not independent, which might cause a selection bias [31]. Among the four contrasts, however, Nested’_(L)_ and Simple’_(S)_ yielded two extremes of the estimates of most factors, without apparent bias among the factors (see Table 1). In addition to both main effects, the percent signal changes in L. F3op/F3t and L. SMG (Figure 4D), averaged across significant voxels, showed a significant length effect within each of Nested’ and Simple’ (paired *t*-test, *P*<0.02; significance level at *α* = 0.025, Bonferroni corrected). Because we used appropriate references of the Conjoined_(L)_ and Conjoined_(S)_, we examined whether likewise *subtracted* estimates of each factor (e.g., DoM for Nested’_(L)_; see Table 1) directly explained parametric modulation of activations in the four contrasts of Nested’_(L)_, Nested’_(S)_, Simple’_(L)_, and Simple’_(S)_. The signal changes in L. F3op/F3t and L. SMG indeed correlated exactly in a step-wise manner with the parametric models of DoM [3, 1, 1, 0] and “DoM+number of Search” [3, 1, 0, –1], respectively.

**Table 4:** Regions related to the sentence conditions and/or string conditions.

Contrast	Brain region	BA	Side	*x*	*y*	*z*	*Z* Value	Voxels
Nested’_(L)_>Simple’_(S)_	F3op/F3t	44/45	L	–45	18	18	4.8	1
	SMG	40	L	–42	–45	42	4.8	2
Nested”>Reverse”	F3op/F3t	44/45	L	–51	24	24	5.8	5
	ACC	6/8/32	M	–3	18	45	5.2	1
	SMG	40	L	–39	–45	42	5.7	27
			R	39	–48	54	4.9	2
	Cerebellum, lobule VI	–	R	27	–69	–24	4.9	1
Nonmatching – Matching: Sentence	F3op/F3t	44/45	R	54	18	30	5.2	14
	LPMC/F3op	6/44	L	–45	9	30	4.8	1
	ACC	6/8/32	M	6	27	42	6.9	52
Nonmatching – Matching: String	F3op/F3t	44/45	R	54	18	30	5.3	21
			R	39	18	33	4.7	1
	SMG	40	R	42	–30	48	5.0	2
Reverse”	LPMC	6	R	27	–9	51	4.7	1

Next we examined how well activations in L. F3op/F3t and L. SMG correlated with DoM and other factors. All contrasts of Nested’_(L)_, etc. predicted that activations should be exactly zero when a factor produced no effect or load relative to the Conjoined. We thus adopted a no-intercept model, in which percent signal changes of each region were fitted with a single (thus minimal) scale parameter to a model of each factor using its subtracted estimates. For the four contrasts, a least-squares method was used to minimize residual sum of squares (RSS) for the four fitted values (i.e., four estimates multiplied by the fitting scale) against corresponding signal changes averaged across participants (Table 5). Among a number of parametric models tested, the model of DoM for L. F3op/F3t, as well as that of “DoM+number of Search” for L. SMG, produced by far the least RSS (≤0.0020) and largest coefficient of determination (*r* ^2^) (≥0.97). Goodness of fit was further evaluated for each model by using a one-sample *t*-test (significance level at *α* = 0.0125, Bonferroni corrected) between the fitted value for each contrast and individual activations. The model of DoM for L. F3op/F3t, as well as that of “DoM+number of Search” for L. SMG, produced no significant deviation for the four contrasts (one-sample *t*-test, *P*≥0.17). For L. SMG, the second-best model was DoM (RSS = 0.0063, *r* ^2^ = 0.92, and its smallest *P* = 0.013 was marginal). To further take account of interindividual variability, we fitted “linear mixed-effects models” with individual activations (Table 5), and found that the models of DoM and “DoM+number of Search” were by far more likely for L. F3op/F3t and L. SMG, respectively.

**Table 5:** Fittings and likelihood of various models tested.

L. F3op/F3t	Factor	RSS	*r* ^2^	*P* values for four contrasts	Log-likelihood	Likelihood ratio
	*DoM	0.0007	0.99	0.17, 0.92, 0.97, 0.99	65.0	1.0
	DoM+No. of Search	0.0065	0.88	0.0035, 0.064, 0.63, 0.88	59.2	3.1×10^–3^
	No. of Search	0.052	<0.1	<0.0001, 0.018, 0.019, 0.031	33.4	2.0×10^–14^
	No. of Merge	0.053	0	<0.0001, 0.0035, 0.018, 0.17	n/a	n/a
	No. of case markers (*-ga*/-*no*)	0.053	0	<0.0001, 0.0035, 0.018, 0.17	n/a	n/a
	No. of tense markers (*-ru*/-*ta*)	0.0067	0.87	0.0035, 0.17, 0.32, 0.56	59.7	4.8×10^–3^
	Degree of nesting	0.010	0.80	0.0035, 0.018, 0.17, >0.99	57.1	3.7×10^–4^
	Degree of self-embedding	0.015	0.71	0.0035, 0.0075, 0.019, 0.17	53.3	8.7×10^–6^
	No. of nodes	0.015	0.72	0.0050, 0.0082, 0.018, 0.17	53.7	1.2×10^–5^
	Depth of postponed symbols	0.053	0	<0.0001, 0.0035, 0.018, 0.17	n/a	n/a
	Integration costs	0.0066	0.88	0.0017, 0,15, 0.48, 0.53	59.0	2.5×10^–3^
	Storage costs	0.014	0.74	<0.0001, 0.024, 0.83, 0.85	53.8	1.3×10^–5^
	Syntactic interference	0.0067	0.87	0.0035, 0.17, 0.32, 0.56	59.7	4.8×10^–3^
	Positional similarity	0.0055	0.90	0.051, 0.12, 0.17, 0.19	60.1	7.8×10^–3^
	Memory span	0.0066	0.88	0.0017, 0,15, 0.48, 0.53	59.0	2.5×10^–3^
	Counting	0.017	0.67	0.0003, 0.0013, 0.035, 0.72	50.8	7.0×10^–7^
	No. of encoding	0.051	<0.1	<0.0001, 0.014, 0.018, 0.12	32.9	1.2×10^–14^
	Memory span+counting	0.0099	0.81	0.0007, 0.035, 0.15, 0.76	55.5	7.9×10^–5^
	Memory span+No. of encoding	0.015	0.72	<0.0001, 0.10, 0.46, 0.59	52.5	3.6×10^–6^
**L. SMG**	**Factor**	**RSS**	***r*** **^2^**	***P*** ** values for four contrasts**	**Log-likelihood**	**Likelihood ratio**
	DoM	0.0063	0.92	0.013, 0.083, 0.44, 0.49	58.8	0.079
	*DoM+No. of Search	0.0020	0.97	0.22, 0.30, 0.42, 0.62	61.4	1.0
	No. of Search	0.075	<0.1	<0.0001, 0.0061, 0.045, 0.090	23.6	3.8×10^–17^
	No. of Merge	0.076	0	<0.0001, 0.0061, 0.013, 0.22	n/a	n/a
	No. of case markers (*-ga*/-*no*)	0.076	0	<0.0001, 0.0061, 0.013, 0.22	n/a	n/a
	No. of tense markers (*-ru*/-*ta*)	0.0079	0.90	0.013, 0.023, 0.22, 0.34	55.9	4.1×10^–3^
	Degree of nesting	0.0088	0.88	0.0061, 0.013, 0.22, >0.99	55.5	2.8×10^–3^
	Degree of self-embedding	0.023	0.69	0.0002, 0.0018, 0.013, 0.22	45.5	1.2×10^–7^
	No. of nodes	0.033	0.56	0.0004, 0.0005, 0.0061, 0.013	40.1	6.0×10^–10^
	Depth of postponed symbols	0.076	0	<0.0001, 0.0061, 0.013, 0.22	n/a	n/a
	Integration costs	0.021	0.72	0.0001, 0.014, 0.028, 0.18	46.3	2.7×10^–7^
	Storage costs	0.032	0.58	<0.0001, 0.0014, 0.084, 0.49	40.3	7.1×10^–10^
	Syntactic interference	0.0079	0.90	0.013, 0.023, 0.22, 0.34	55.9	4.1×10^–3^
	Positional similarity	0.020	0.73	0.0039, 0.0052, 0.013, 0.029	47.6	1.0×10^–6^
	Memory span	0.021	0.72	0.0001, 0.014, 0.028, 0.18	46.3	2.7×10^–7^
	Counting	0.041	0.46	<0.0001, <0.0001, 0.0039, 0.77	35.6	6.2×10^–12^
	No. of encoding	0.076	<0.1	<0.0001, 0.0061, 0.017, 0.16	22.5	1.4×10^–17^
	Memory span+counting	0.028	0.63	<0.0001, 0.0018, 0.0086, 0.44	41.3	1.9×10^–9^
	Memory span+No. of encoding	0.011	0.85	0.0034, 0.051, 0.13, 0.81	52.1	9.7×10^–5^

Percent signal changes in L. F3op/F3t and L. SMG were fitted with a single scale parameter to a model of each factor using its subtracted estimates (Table 1) for the four contrasts of Nested’_(L)_, Nested’_(S)_, Simple’_(L)_, and Simple’_(S)_. The *P* values for the *t*-tests are shown in ascending order. Note that the models of DoM and “DoM+number of Search” (with an asterisk) resulted in the best fit for L. F3op/F3t and L. SMG, respectively, i.e., with the least residual sum of squares (RSS), largest coefficient of determination (*r* ^2^), and larger *P* values. The likelihood of models with all null estimates was incalculable (n/a). A likelihood ratio is the ratio of each model’s likelihood to the best model’s likelihood. The best models of DoM and “DoM+number of Search” for L. F3op/F3t and L. SMG, respectively, were by far more likely than the other models.

Next, we examined whether the selective activation in these regions was replicated even after controlling both matching orders and symbol orders (e.g., N_2_ N_1_ V_1_ V_2_ and A_2_ A_1_ B_1_ B_2_) between the Nested and Reverse, i.e., in Nested”>Reverse” combining the short and long stimuli. This contrast indeed resulted in significant activation in L. F3op/F3t and L. SMG (Figure 4E and Table 4). In both regions, the signal changes in Reverse” were not significantly different from 0 (one-sample *t*-test, *P*>0.1) (Figure 4F). Moreover, the models of DoM and “DoM+number of Search” were also consistent with the signal changes in both Nested” and Reverse” (Table 2). The number of encoding might explain the results of Figure 4F, but its estimates cannot consistently explain the results of Figure 4D.

### Effective and Anatomical Connectivity between L. F3op/F3t and L. SMG

Based on these results, we modeled effective connectivity between L. F3op/F3t and L. SMG by using DCM. Our interest was to identify the direction of the connectivity modulated by the Nested condition with largest DoM among all conditions, and the models were grouped into three “modulatory families”: families *A*, *B*, and *C*, corresponding to the modulation for the bottom-up connection from L. SMG to L. F3op/F3t, for the top-down connection from L. F3op/F3t to L. SMG, and for both connections, respectively. Here we assumed intrinsic, i.e., task-independent, bidirectional connections. Each family was composed of three “input models” as regards the regions receiving driving inputs (see Figure S4 for all DCM models tested). Using a random-effects Bayesian model selection (BMS), we found that the family *A* was the most likely family (expected probability = 0.66, exceedance probability = 0.85) (Figure 4G and 4H). According to a second BMS for the input models within the family *A*, the model *A1*, in which L. F3op/F3t received driving inputs, was the best and highly probable model (expected probability = 0.77, exceedance probability = 0.95). For this particular model, we further tested whether the parameter estimates were significantly different from zero. The intrinsic connection from L. F3op/F3t to L. SMG was significantly positive [+0.22; one-sample *t*-test, *t*(17) = 4.8, *P*<0.0002] (significance level at *α* = 0.025, Bonferroni corrected within a parameter class of intrinsic connections) (Figure 4I), indicating that this top-down connection was consistent among the participants. The modulatory effect for the bottom-up connection was inhibitory [–0.17; *t*(17) = 1.4, *P* = 0.17], though it did not reach the significance level.

To further confirm the anatomical plausibility of the network between L. F3op/F3t and L. SMG revealed by DCM, we used DTI with a probabilistic tractography. Seed masks were set in the pair of L. F3op/F3t and L. SMG, both of which were significantly activated in Nested’_(L)_>Simple’_(S)_. We identified a single continuous cluster of the left SLF/AF that connected these regions (cluster size, 3,189 mm^3^), together with much smaller clusters or islands (Figure 4J). Moreover, the left SLF/AF was consistently observed in all participants.

### Modulation of the Right Frontal Activations by Nonlinguistic Factors

**Figure 5:**
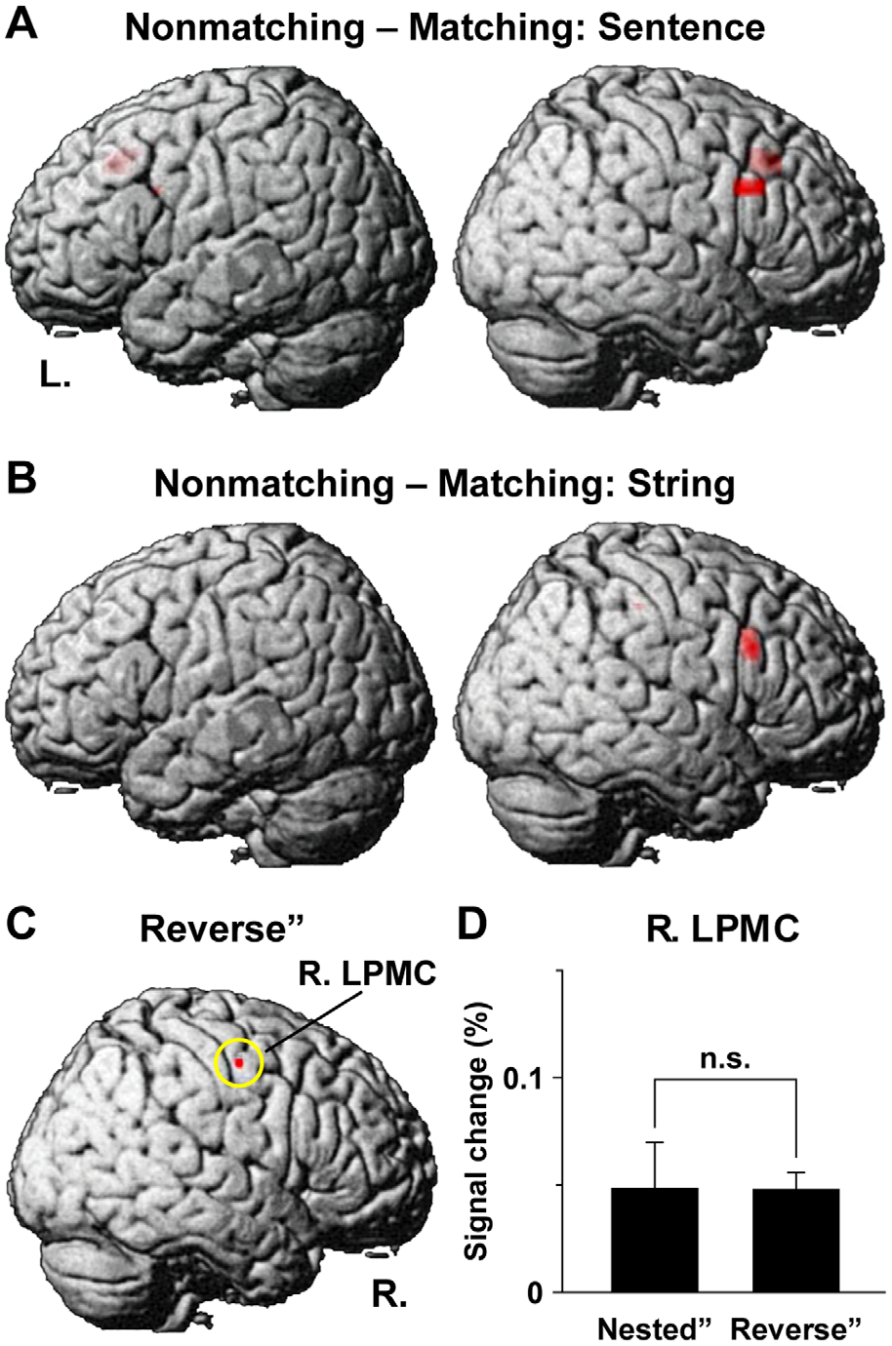
Modulation of the right frontal activations by nonlinguistic factors. One-sample *t*-tests were used for the contrasts indicated. (A) Regions identified by [Nonmatching – Matching] under the sentence conditions, related to error-related factors. Note the right-dominant activation, especially in R. F3op/F3t. (B) Regions identified by [Nonmatching – Matching] under the string conditions. (C) Regions identified by Reverse”. This contrast revealed the difference in matching orders (e.g., A_2_ A_1_ B_1_ B_2_ vs. A_1_ A_2_ B_1_ B_2_). Note the significant activation in R. LPMC. (D) The percent signal changes in R. LPMC, which was consistent with the equivalent estimates of memory span (see Table 2).

We further examined the involvement of any error-related factors, which were residual factors that might induce cortical activation or deactivation. It should be noted that the factors listed in Tables 1 and 2 were equivalent between the matching and nonmatching stimuli. The [Nonmatching – Matching] contrast under either the sentence conditions (i.e., [Nested+Simple+Conjoined]) or the string conditions (i.e., [Reverse+Same]) consistently resulted in right-dominant activation, especially in R. F3op/F3t (Figure 5A and 5B), which was in accordance with the same demand of the matching task (Figure 1C and 1D). Other significantly activated regions were L. LPMC/F3op and ACC under the sentence conditions, as well as R. SMG under the string conditions (Table 4). As regards the [Matching – Nonmatching] contrast, no significant activation was seen under sentence or string conditions.

We also examined the activation in Reverse” for the effect of matching orders (e.g., A_2_ A_1_ B_1_ B_2_ vs. A_1_ A_2_ B_1_ B_2_; Figures 1B and 2B). The significant activation was observed only in R. LPMC (Figure 5C and Table 4), which suggested that activations could indeed be estimated by one and only non-null factor of memory span in Reverse” (Table 2). In Nested”, the signal changes in R. LPMC were also significant (one-sample *t*-test, *P*<0.05), which were not significantly different between Nested” and Reverse” (paired *t*-test, *P* = 0.98) (Figure 5D). This result was consistent with the equivalent estimates of memory span between Nested” and Reverse”. It should be noted that R. LPMC activation was also observed for the main effects of condition and length (Figure 4A and 4B), which probably reflected the factor of memory span.

## Discussion

By employing a novel paradigm to directly contrast jabberwocky sentences (Nested, Simple, and Conjoined) with letter strings (Reverse and Same) (Figures 1 and 2), we obtained four striking results. First, we found that DoM was indeed a key syntactic factor that could account for syntax-selective activations in L. F3op/F3t and L. SMG, localized by the Nested’_(L)_>Simple’_(S)_ contrast (Figure 4C and 4D). By constructing a model of each syntactic, other linguistic, or nonlinguistic factor using its estimates (Table 1), we demonstrated that the models of DoM and “DoM+number of Search” were the best to explain L. F3op/F3t and L. SMG activations, respectively (Table 5). Secondly, by directly contrasting jabberwocky sentences with letter strings, i.e., Nested”>Reverse”, we showed that the selective activation in L. F3op/F3t and L. SMG, which was consistent with the involvement of the syntactic factors demonstrated above, was replicated irrespective of identical matching orders and symbol orders (e.g., N_2_ N_1_ V_1_ V_2_ and A_2_ A_1_ B_1_ B_2_ for the Nested and Reverse, respectively) (Figure 4E and 4F). This point is particularly important, because temporal order-related or memory-related factors have often been confused with differences in structure or grammar type. Our results strongly support that syntactic structures are recursively constructed when well-formed sentences are given. Thirdly, by using DCM, we found that the model with a inhibitory modulatory effect for the bottom-up connectivity from L. SMG to L. F3op/F3t, and with driving inputs to L. F3op/F3t, was the best one (Figure 4G and 4H). For this best model, the top-down connection from L. F3op/F3t to L. SMG was significantly positive (Figure 4I). By using DTI, we also confirmed that the left dorsal pathway of SLF/AF consistently connected these two regions (Figure 4J). These results suggest that there is a transmission of information about DoM through this specific dorsal pathway. Lastly, we established that nonlinguistic order-related and error-related factors significantly activated mostly right frontal regions. The difference in memory span significantly modulated R. LPMC activation in Reverse”, suggesting that this region plays a major role in tracking matching orders (Figure 5C and 5D), while error-related factors in [Nonmatching – Matching] consistently modulated R. F3op/F3t activation under both sentence and string conditions (Figure 5A and 5B). In summary, these results indicate that the identified network of L. F3op/F3t and L. SMG subserves the calculation of DoM in recursively merged sentences, and that R. LPMC monitors memory span to drive a memory-maintenance system. If multiple factors, such as the number of nodes, memory span, etc., are equally plausible to explain activations, then a superordinate concept, such as “syntactic complexity”, can be a more useful factor than individual factors. However, in the present experiment, the minimal factor of DoM *sufficiently* explained the activation pattern observed, while other factors were by far less likely (see Table 5). Therefore, syntactic complexity was restricted and replaced by DoM as a more fundamental concept, just like the historical development from “gene” to DNA.

It remains a central issue in cognitive sciences whether or not the faculty of language is also shared by animals. Animals have been thus tested with regular symbolic sequences such as A*^n^* B*^n^* (*n* ≥2; i.e., AABB, AAABBB, …) and (AB)*^n^* (*n* ≥2; i.e., ABAB, ABABAB, …), which differ in *symbol order*. In an animal study, songbirds were trained to discriminate patterns of A*^n^* B*^n^* and (AB)*^n^* in more than ten thousand trials [32]. However, this learning can be achieved by a counting strategy alone [33]. There is also a recent report that songbirds seemed to discriminate strings with or without nesting [34], but this learning can be achieved by simply remembering partial strings [35]. Along the line of contrasting A*^n^* B*^n^* and (AB)*^n^*, fMRI studies have tested participants with different symbolic sequences, such as A_2_ A_1_ B_1_ B_2_ versus A_1_ B_1_ A_2_ B_2_, which also differ in *matching order* [36]. However, the difference in activation patterns can be simply explained by differences in any factors associated with matching orders and symbol orders, i.e., temporal order-related factors. It was thus necessary to completely control these general factors when extracting any syntactic factors from a number of cognitive factors involved in actual symbol processing.

Our finding that L. F3op/F3t subserves the syntactic computation further extends the functional specialization of this region reported previously [14], [18]–[20]. Some previous fMRI studies have interpreted L. F3op/F3t activation as reflecting temporal order-related or memory-related factors [37], [38]. However, these previous studies contrasted hierarchically complex sentences with simpler sentences, while it is clear that syntactic factors, including DoM, were also involved. Moreover, the previously reported modulation of the L. F3op/F3t activation by scrambling word orders [3] can be consistently explained by DoM, because scrambling requires “movements” of NPs to higher nodes by applying more Merge operations, thus increasing DoM. The size of linguistic constituents also correlates with DoM, especially when the number of left/right branches was increased as in the case of Pallier et al. (2011) [4]. In the present study, we characterized the neural substrates of syntactic computation by segregating a number of possible factors, and demonstrated that the exact activations in L. F3op/F3t can be used to calculate DoM. Indeed, each structure of our jabberwocky sentences was uniquely represented by DoM, together with the numbers of Merge and Search (see Table 1).

A previous fMRI study involving the implicit learning of an artificial regular grammar has reported that the “ungrammatical – grammatical” contrast for symbol sequences activated L. F3op/F3t, suggesting that such activation was due to artificial syntactic violations among any error-related factors [39]. However, this result may not depend on the presence of errors themselves, but on other rule-related processes associated with error-correction, etc. In contrast, we have previously demonstrated that an explicit syntactic decision enhanced L. F3op/F3t activation under *both* grammatical and ungrammatical conditions [17]. On the other hand, a recent fMRI study has compared nested and branching constructions, suggesting that activation in the bilateral posterior superior temporal cortex reflects an integration of lexico-semantic and syntactic information [40]. However, as regards this previous result, the effects of semantic factors were inevitably confounded with any structural processing, because real German sentences were used as stimuli in that study. Furthermore, according to our paradigm, the temporal cortex in neither of the hemispheres showed any significant activation for the Nested (Figure 4). It was thus quite important to verify that activation in L. F3op/F3t, but not in the temporal cortex, is indeed crucial for syntactic processing.

In the present study, we found that L. SMG activations were modulated by “DoM+number of Search”. Consistent with the suggested role of L. AG/SMG for vocabulary knowledge or lexical processing [21], [22], the number of Search is likely to induce such a modulation, in the sense that Search assigns a specific feature that can be linked with morphosyntactic changes. The Japanese language happens to lack the agreement of grammatical features, but it is nevertheless equipped with the general Search procedure attested for various phenomena in the language [8]. Our results suggest that Search actually applied to a subject-verb pair of a jabberwocky sentence in the present paradigm, where the relevant features (vowels here) are experimentally “inserted”. It should also be noted in this connection that the Japanese language exhibits a phenomenon called “honorification” (the case of an honored person and the form of honorifics on verbs optionally match) [41], [42], in which Search assigns such features as honorifics. Our previous fMRI study using an honorification judgment task reported activation in L. F3op/F3t and L. LPMC, as well as in the L. inferior parietal gyrus and L. AG [43], which is consistent with activation in L. AG/SMG in the present study (Tables 3 and 4).

Our DCM and DTI results further indicate that L. SMG activations reflecting DoM mirrored a top-down influence from L. F3op/F3t through the left dorsal pathway of SLF/AF. A recent DCM study with a picture-sentence matching task has suggested that L. F3op/F3t received driving inputs [44], which was consistent with our DCM results. Moreover, our previous studies revealed that the functional connectivity between L. F3t/F3O (pars orbitalis) and L. AG/SMG was selectively enhanced during sentence processing [45], and that L. AG/SMG was also activated during the identification of correct past-tense forms of verbs, probably reflecting an integration of syntactic and vocabulary knowledge [46]. Considering the role of L. AG/SMG in lexical processing, the Search operation based on DoM would be essential in assigning relevant features to the syntactic objects derived from lexical items.

In [Nonmatching – Matching], R. F3op/F3t was consistently activated under both sentence and string conditions (Figure 5A and 5B), whereas L. LPMC/F3op, ACC, or R. SMG were activated under either condition. These four regions were also activated in Nested’>Simple’, and in Long>Short while combining Nested’ and Simple’; the ACC and R. SMG were activated in Nested”>Reverse” as well. It appears likely that a part of the activation in these four regions reflects error-related factors including the detection and correction of errors, which would be more demanding with the Nested, as well as in the Long>Short contrast. Because L. LPMC has been known to selectively subserve syntactic processing [15], [19], [47], a weak activation in L. LPMC/F3op only under the sentence conditions may reflect the confirmation of sentence constructions when confronted with nonmatching stimuli. On the other hand, it has been suggested that the dorsal ACC plays a major role during conflict monitoring during a highly demanding task, e.g., a Stroop task [48]. Our recent magnetoencephalography study also suggested that the anterior portion of the ACC is a candidate region for monitoring syntactically anomalous sentences [49]. Moreover, previous studies on a response inhibition, typically tested with a No-go task, suggested that R. F3op/F3t, ACC, and R. SMG were also involved in monitoring anomalous stimuli [50]. In contrast to these factors that activated mostly right and medial regions, it is noteworthy that the syntactic factors clearly activated the left frontal and parietal regions.

Any factors associated with matching orders and symbol orders might influence activation in the language areas, but we clearly showed that R. LPMC was activated in Reverse” (Figure 5C) for the effect of memory span related to matching orders. The study of real German sentences also reported activation in the right dorsal premotor area for the contrast of nested vs. branching constructions [40], but the right dorsal premotor area was not the same region as R. LPMC in the present study. In this German study, memory span was controlled by the insertion of some words, while matching orders and symbol orders still differed, and thus factors other than memory span were inevitably introduced to interpret the right dorsal premotor activation. The identification of critical factors in language processing thus inevitably depends on an experimental design that involves an effective contrast of conditions. One promising direction of research is to further clarify activations modulated by other linguistic and nonlinguistic factors, which may eventually make possible the elucidation of all aspects of linguistic information in the human brain.

## Supporting Information

Figure S1 **Application of other structure-based models to sentences with complex structures, I.** (A) The digits shown in red and blue denote “degree of nesting” and “degree of self-embedding”, respectively. Nested and self-embedded constructions occur within sentences (Ss). Note that each shortest “zigzag path” counts one for the degree of nesting or self-embedding. For the Nested_(L)_, S_1_ dominates [N_2_ S_2_ V_2_], and S_0_ in turn dominates [N_3_ S_1_ V_3_], i.e., [N_3_[N_2_ S_2_ V_2_]V_3_]; the degree of nesting or self-embedding is thus two (the number of blue dots minus one). For the Simple_(L)_, both of (NN)N_1_ and N(NN_1_) yield the same maximum degree of nesting or self-embedding for an entire sentence. (B) The digits shown in red denote “number of nodes”.
(TIF)

Figure S2 **Application of other structure-based models to sentences with complex structures, II.** The digits shown in red and blue denote the number of branches from each node and “depth of postponed symbols” [51], respectively. The largest estimate can be obtained by adding together the digits shown in red with circles. For the Simple_(L)_, the largest estimate of “depth of postponed symbols” is obtained, when Vs take a right-branching construction of V_1_(VV_1_). For the Conjoined_(L)_, the depth of postponed symbols is increased by two to reach the rightmost branches, when conjoining three sentences at a multiple-branching node.
(TIF)

Figure S3 **Application of other structure-based models to sentences with complex structures, III.** (A) The digits shown in red and blue denote “integration costs” and “storage costs” [52], respectively. Integration costs are estimated at every stimulus by adding together “new discourse referents” and “structural integrations”. For example, at V_2_ of the Nested_(L)_, N_1_ and V_1_ intervene while making [N_2_[N_1_ V_1_]V_2_] (structural integrations = 2), and one verb completes the input with *-to* or *-te* (storage cost = 1). Note that the estimate of maximum structural integrations in a sentence matches with that of memory span in our paradigm. (B) The digits shown in red and blue denote “syntactic interference” and “positional similarity” [53], respectively. Syntactic interference is estimated at every stimulus by adding together “retroactive interference” and “proactive interference”. For example, at V_2_ of the Nested_(L)_, the attachment of V_2_ to N_2_ suffers from one unit of retroactive interference from N_1_, and from one unit of proactive interference from N_3_ (syntactic interference = 2). There are three adjacent nominative NPs in this sentence (positional similarity = 3).
(TIF)

Figure S4 **The DCM models tested.** We assumed bidirectional connectivity between L. F3op/F3t and L. SMG. The models were grouped into three modulatory families based on the modulations of the connections under the Nested condition: Family *A* (*A1*–*A3*), in which the connection from L. SMG to L. F3op/F3t was modulated, Family *B* (*B1*–*B3*), in which the connection from L. F3op/F3t to L. SMG was modulated, and Family *C* (*C1*–*C3*), in which both connections were modulated. Each family was composed of three “input models” as regards the regions receiving driving inputs.
(TIF)

Table S1 **Examples of short nonmatching stimuli.**
(PDF)

Table S2 **Examples of long nonmatching stimuli.**
(PDF)

Appendix S1 **Theoretical issues.**
(PDF)

Appendix S2 **Detailed information about the stimuli.**
(PDF)

Appendix S3 **Task instructions and training procedures.**
(PDF)

Appendix S4 **MRI data analyses.**
(PDF)
