# Supplementary figures and images for: Syntactic Computation in the Human Brain: The Degree of Merger as a Key Factor
Source: PLoS One. 2013 Feb 20;8(2):e56230. doi: 10.1371/journal.pone.0056230 (PMC3577822; doi:10.1371/journal.pone.0056230)

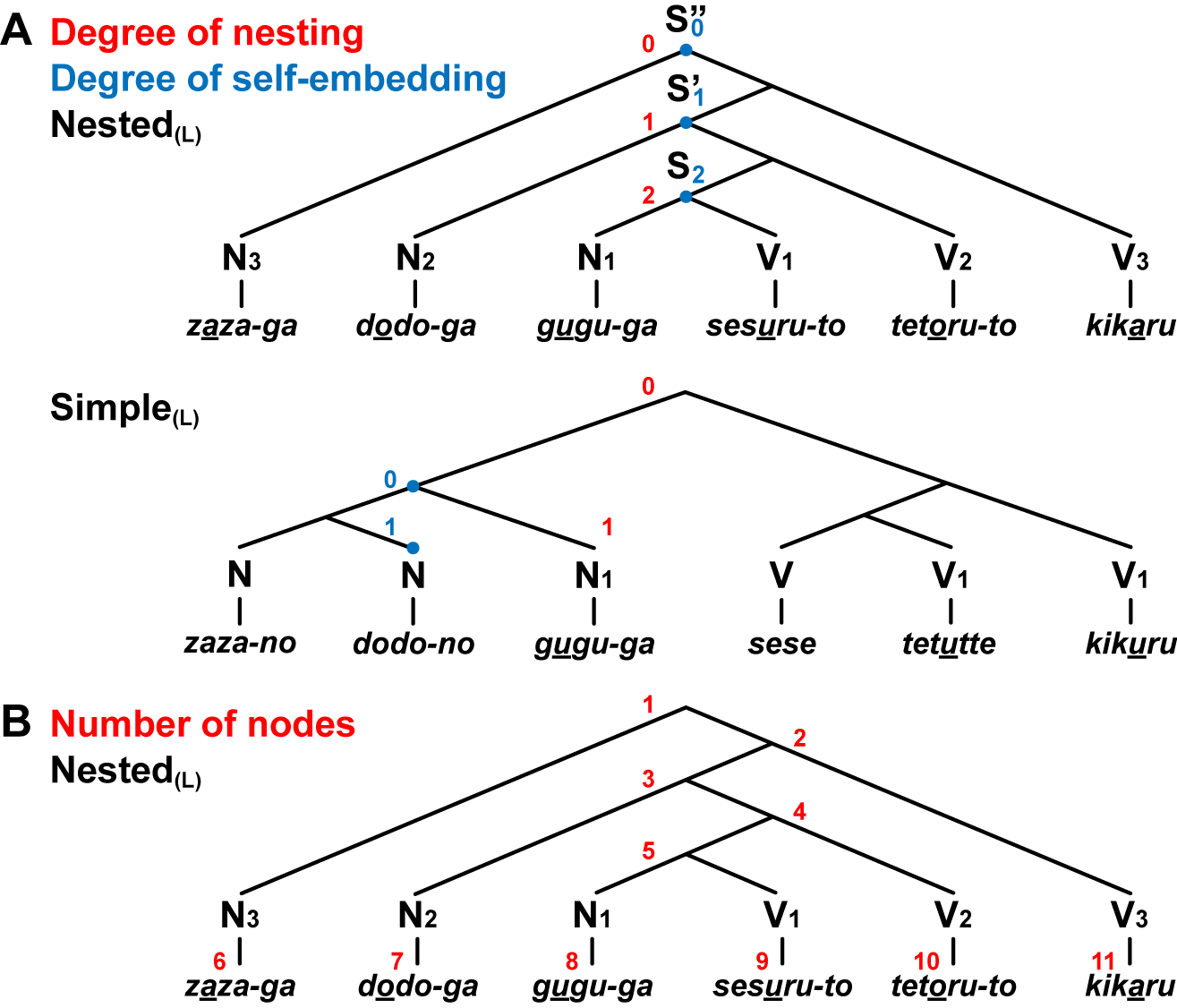

Supplement: Figure S1 — Application of other structure-based models to sentences with complex structures, I. (A) The digits shown in red and blue denote “degree of nesting” and “degree of self-embedding”, respectively. Nested and self-embedded constructions occur within sentences (Ss). Note that each shortest “zigzag path” counts one for the degree of nesting or self-embedding. For the Nested(L), S1 dominates [N2 S2 V2], and S0 in turn dominates [N3 S1 V3], i.e., [N3[N2 S2 V2]V3]; the degree of nesting or self-embedding is thus two (the number of blue dots minus one). For the Simple(L), both of (NN)N1 and N(NN1) yield the same maximum degree of nesting or self-embedding for an entire sentence. (B) The digits shown in red denote “number of nodes”. (TIF) [file pone.0056230.s001.tif]

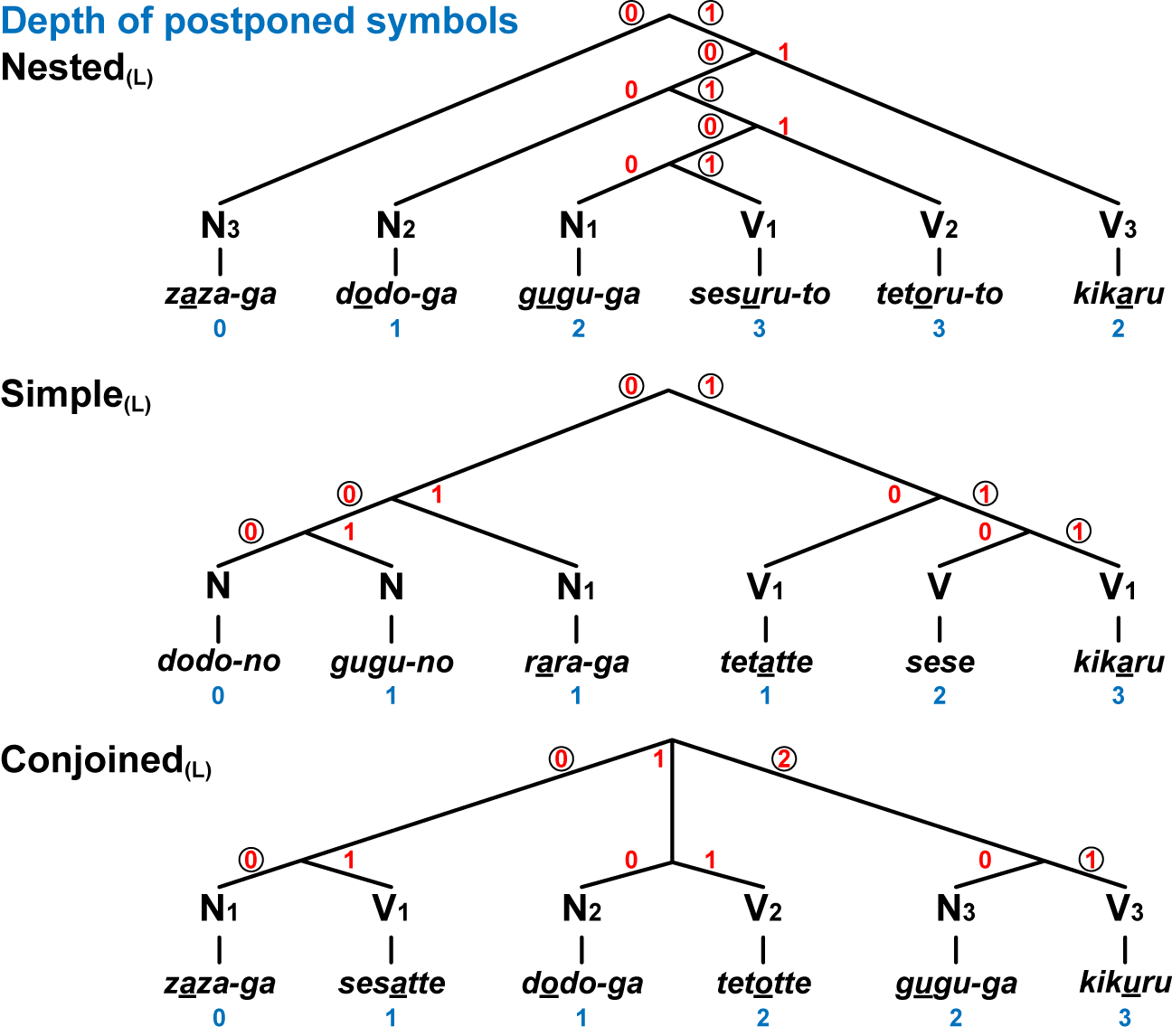

Supplement: Figure S2 — Application of other structure-based models to sentences with complex structures, II. The digits shown in red and blue denote the number of branches from each node and “depth of postponed symbols” [51], respectively. The largest estimate can be obtained by adding together the digits shown in red with circles. For the Simple(L), the largest estimate of “depth of postponed symbols” is obtained, when Vs take a right-branching construction of V1(VV1). For the Conjoined(L), the depth of postponed symbols is increased by two to reach the rightmost branches, when conjoining three sentences at a multiple-branching node. (TIF) [file pone.0056230.s002.tif]

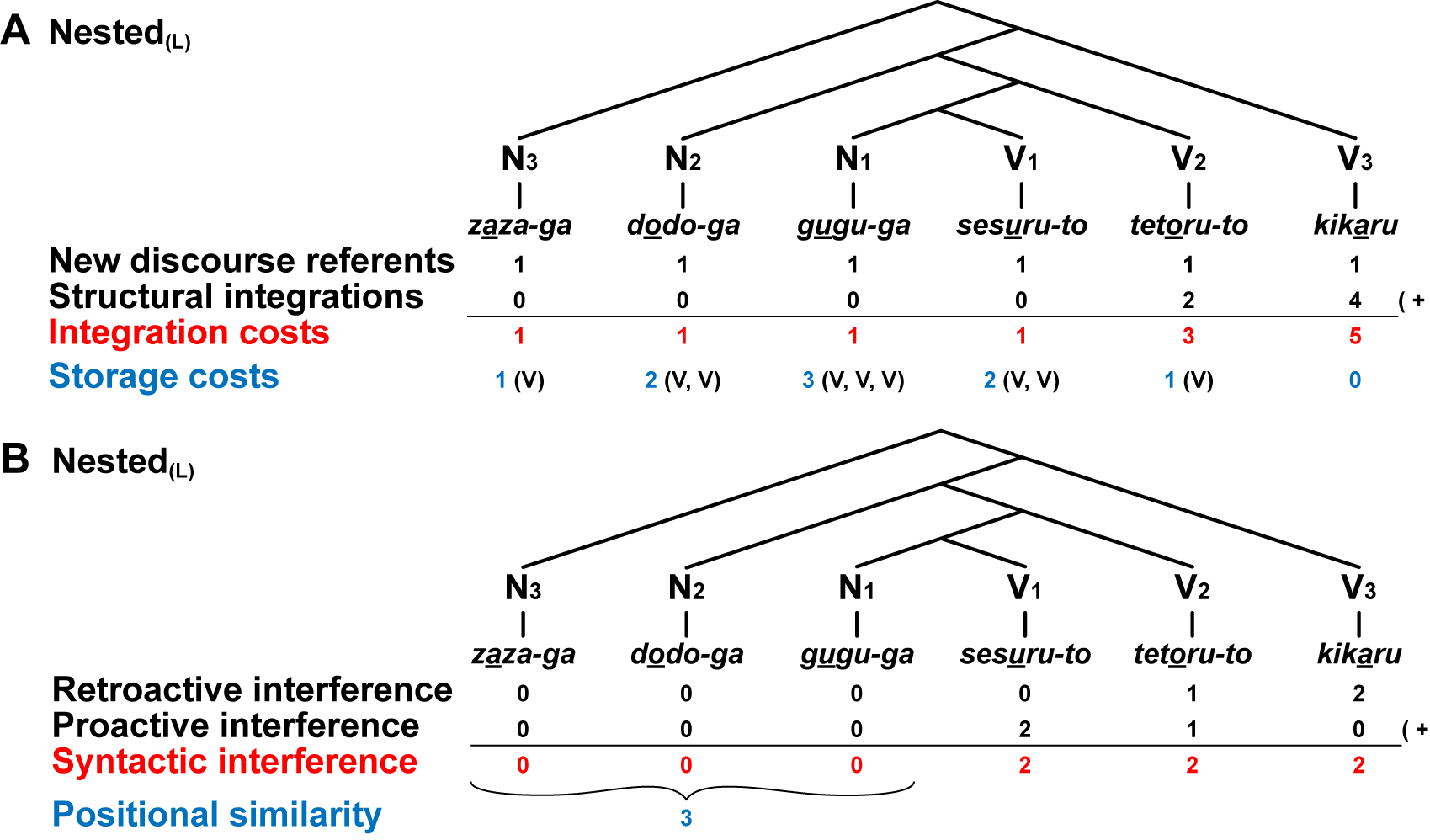

Supplement: Figure S3 — Application of other structure-based models to sentences with complex structures, III. (A) The digits shown in red and blue denote “integration costs” and “storage costs” [52], respectively. Integration costs are estimated at every stimulus by adding together “new discourse referents” and “structural integrations”. For example, at V2 of the Nested(L), N1 and V1 intervene while making [N2[N1 V1]V2] (structural integrations = 2), and one verb completes the input with -to or -te (storage cost = 1). Note that the estimate of maximum structural integrations in a sentence matches with that of memory span in our paradigm. (B) The digits shown in red and blue denote “syntactic interference” and “positional similarity” [53], respectively. Syntactic interference is estimated at every stimulus by adding together “retroactive interference” and “proactive interference”. For example, at V2 of the Nested(L), the attachment of V2 to N2 suffers from one unit of retroactive interference from N1, and from one unit of proactive interference from N3 (syntactic interference = 2). There are three adjacent nominative NPs in this sentence (positional similarity = 3). (TIF) [file pone.0056230.s003.tif]

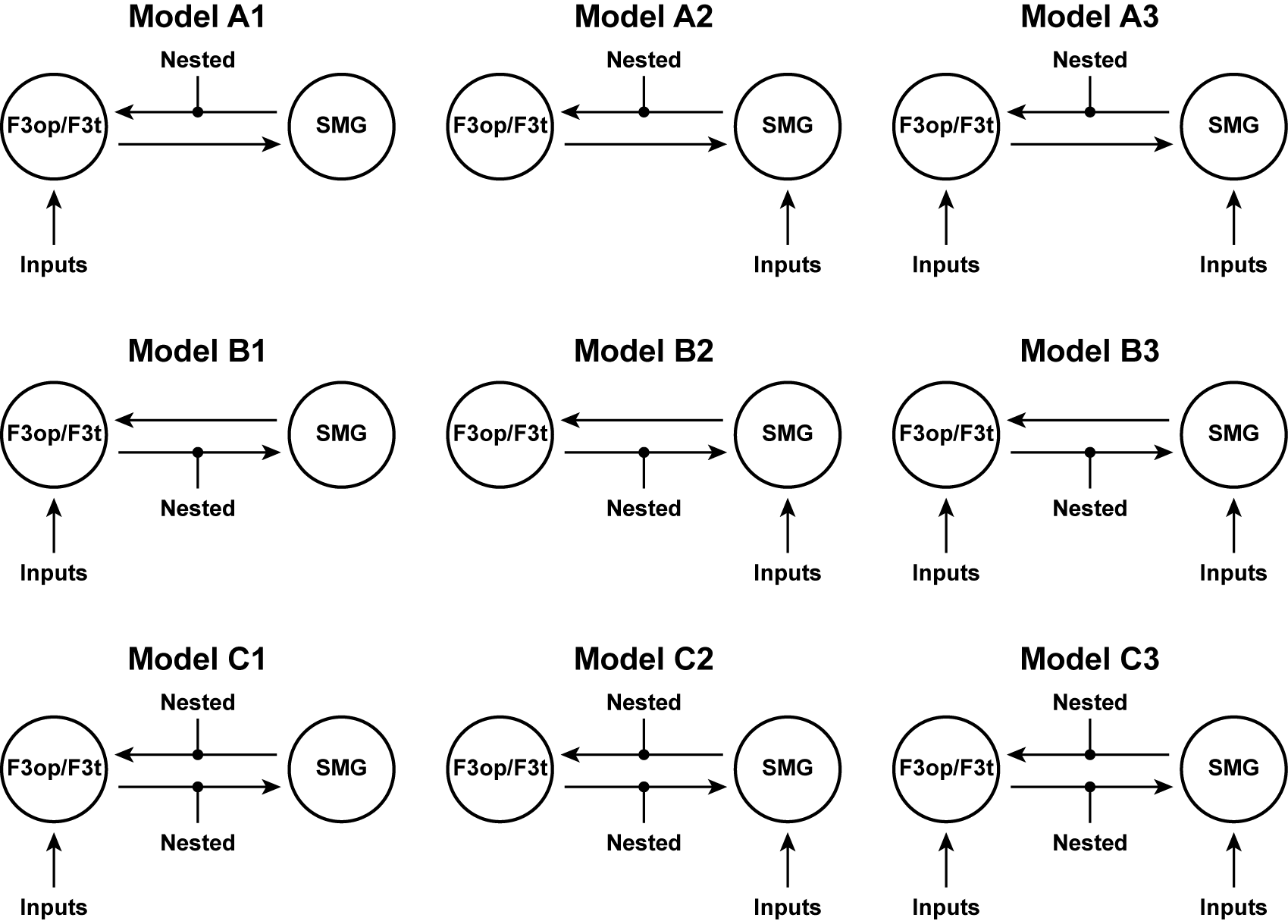

Supplement: Figure S4 — The DCM models tested. We assumed bidirectional connectivity between L. F3op/F3t and L. SMG. The models were grouped into three modulatory families based on the modulations of the connections under the Nested condition: Family A (A1–A3), in which the connection from L. SMG to L. F3op/F3t was modulated, Family B (B1–B3), in which the connection from L. F3op/F3t to L. SMG was modulated, and Family C (C1–C3), in which both connections were modulated. Each family was composed of three “input models” as regards the regions receiving driving inputs. (TIF) [file pone.0056230.s004.tif]
