# Supplementary material for: Syntactic Computation in the Human Brain: The Degree of Merger as a Key Factor
Source: PLoS One. 2013 Feb 20;8(2):e56230. doi: 10.1371/journal.pone.0056230 (PMC3577822; doi:10.1371/journal.pone.0056230)
Supplement: Table S1 — Examples of short nonmatching stimuli. (PDF) [file pone.0056230.s005.pdf]

Syntactic Computation in the Human Brain: The Degree of Merger as a Key Factor

Shinri Ohta, Naoki Fukui, Kuniyoshi L. Sakai

Table S1. Examples of short nonmatching stimuli.

**Condition**	**Error type**	**Stimulus example**
Nested sentence, short	$[N_2[N_1\ V_1]V_2*]$	*mumu-ga rara-ga tetaru-to hihoru*
(Nested$_{(S)}$)	$[N_2[N_1\ V_1*]V_2*]$	*dodo-ga gugu-ga tetoru-to kikaru*
	$[N_2[N_1\ V_1*]V_2]$	*rara-ga mumu-ga sesotta-to kikatta*
Simple sentence, short (Simple$_{(S)}$)	$[(NN_1)\ (VV_1*)]$	*rara-no gugu-ga tete sesatta*
Conjoined sentence, short	$[N_1\ V_1][N_2\ V_2*]$	*rara-ga hihatte gugu-ga tetoru*
(Conjoined$_{(S)}$)	$[N_1\ V_1*][N_2\ V_2*]$	*yoyo-ga hihutte rara-ga sesotta*
	$[N_1\ V_1*][N_2\ V_2]$	*gugu-ga tetotte zaza-ga hiharu*
Reverse-order string, short	$A_2\ A_1\ B_1\ B_2*$	*nododo rukiku rukiku donodo*
(Reverse$_{(S)}$)	$A_2\ A_1\ B_1*\ B_2*$	*gayoyo settasa sasseta gadodo*
	$A_2\ A_1\ B_1*\ B_2$	*mugamu sessota kittako mugamu*
Same-order string, short	$A_1\ A_2\ B_1\ B_2*$	*ruteta gugagu ruteta yogayo*
(Same$_{(S)}$)	$A_1\ A_2\ B_1*\ B_2*$	*yonoyo rusesu donodo rususe*
	$A_1\ A_2\ B_1*\ B_2$	*norara kuruki noyoyo kuruki*

For each condition, nonmatching stimuli with errors in different positions are listed in descending order of frequency. Under the sentence conditions, Vs with asterisks represent matching errors (underlined vowels in stimulus examples here; no underline in the real stimuli). Under the string conditions, Bs with asterisks represent matching errors (underlined letter strings in stimulus examples here; no underline in the real stimuli).
