# Supplementary material for: Syntactic Computation in the Human Brain: The Degree of Merger as a Key Factor
Source: PLoS One. 2013 Feb 20;8(2):e56230. doi: 10.1371/journal.pone.0056230 (PMC3577822; doi:10.1371/journal.pone.0056230)
Supplement: Table S2 — Examples of long nonmatching stimuli. (PDF) [file pone.0056230.s006.pdf]

Syntactic Computation in the Human Brain: The Degree of Merger as a Key Factor

Shinri Ohta, Naoki Fukui, Kuniyoshi L. Sakai

Table S2. Examples of long nonmatching stimuli.

Condition	Error type	Stimulus example
$\text{Nested}_{(L)}$	$[N_3[N_2[N_1\ V_1]V_2]V_3*]$	*mumu-ga zaza-ga yoyo-ga kikotta-to hihatta-to tetatta*
	$[N_3[N_2[N_1\ V_1]V_2*]V_3]$	*dodo-ga rara-ga mumu-ga hihuru-to teturu-to kikoru*
	$[N_3[N_2[N_1\ V_1]V_2*]V_3*]$	*dodo-ga mumu-ga zaza-ga tetaru-to sesoru-to hihuru*
	$[N_3[N_2[N_1\ V_1*]V_2]V_3*]$	*rara-ga mumu-ga yoyo-ga hiharu-to teturu-to sesoru*
$\text{Simple}_{(L)}$	$[((NN)N_1)\ ((VV_1)V_1*)]$	*rara-no gugu-no yoyo-ga kiki sesotte teturu*
	$[((NN)N_1)\ ((VV_1*)V_1)]$	*gugu-no zaza-no dodo-ga kiki tetatte sesoru*
	$[((NN)N_1)\ ((VV_1*)V_1*)]$	*yoyo-no rara-no mumu-ga tete hihotte kikotta*
$\text{Conjoined}_{(L)}$	$[N_1\ V_1][N_2\ V_2][N_3\ V_3*]$	*dodo-ga tetotte mumu-ga sesutte zaza-ga hihoru*
	$[N_1\ V_1][N_2\ V_2*][N_3\ V_3]$	*gugu-ga kikutte zaza-ga tetotte dodo-ga sesoru*
	$[N_1\ V_1][N_2\ V_2*][N_3\ V_3*]$	*zaza-ga sesatte yoyo-ga kikutte gugu-ga tetotta*
	$[N_1\ V_1*][N_2\ V_2][N_3\ V_3*]$	*mumu-ga sesotte rara-ga kikatte dodo-ga hihutta*
$\text{Reverse}_{(L)}$	$A_3\ A_2\ A_1\ B_1\ B_2\ B_3*$	*gazaza rusose gunogu gunogu rusose nozaza*
	$A_3\ A_2\ A_1\ B_1\ B_2*\ B_3*$	*ragara hiruhu gayoyo gayoyo huruhi gazaza*
	$A_3\ A_2\ A_1\ B_1\ B_2*\ B_3$	*serusa gugagu hohiru hohiru nogugu serusa*
	$A_3\ A_2\ A_1\ B_1*\ B_2\ B_3*$	*gunogu ruteta nododo noyoyo ruteta gugagu*
$\text{Same}_{(L)}$	$A_1\ A_2\ A_3\ B_1\ B_2\ B_3*$	*hiruho gunogu haruhi hiruho gunogu hahiru*
	$A_1\ A_2\ A_3\ B_1\ B_2*\ B_3*$	*dogado rutetu zagaza dogado suruse zanoza*
	$A_1\ A_2\ A_3\ B_1\ B_2*\ B_3$	*kattaki yonoyo tutetta kattaki nododo tutetta*
	$A_1\ A_2\ A_3\ B_1*\ B_2\ B_3*$	*noyoyo tahihha munomu nododo tahihha mugamu*

For the $\text{Simple}_{(L)}$, there were also other error types, i.e., $V_1*(VV_1*)$, $V_1(VV_1*)$, and $V_1*(VV_1)$. For the $\text{Nested}_{(L)}$ and $\text{Conjoined}_{(L)}$, we included nonmatching stimuli with a maximum variety of vowels in Vs, as shown here; for the $\text{Simple}_{(L)}$, we included nonmatching stimuli with a least variety of vowels in Vs. Therefore, the strategy of noting the variety of vowels was not effective. Under all conditions, there were more variations in error for the long than short stimuli.
