# Supplementary material for: Syntactic Computation in the Human Brain: The Degree of Merger as a Key Factor
Source: PLoS One. 2013 Feb 20;8(2):e56230. doi: 10.1371/journal.pone.0056230 (PMC3577822; doi:10.1371/journal.pone.0056230)
Supplement: Appendix S1 — Theoretical issues. (PDF) [file pone.0056230.s007.pdf]

Syntactic Computation in the Human Brain: The Degree of Merger as a Key Factor

Shinri Ohta, Naoki Fukui, Kuniyoshi L. Sakai

Appendix S1 Theoretical issues

Theoretical Background

Sentences with different constructions have been previously discussed in terms of the acceptability of sentences [cf. [1], p. 12].

(i) nested constructions,
(ii) self-embedded constructions,
(iii) multiple-branching constructions,
(iv) left-branching constructions, and
(v) right-branching constructions

The nested constructions are created by *centrally* embedding a phrase within another phrase (with some non-null element to its left and some non-null element to its right), and the self-embedded constructions are the special case of nested constructions when nesting occurs within the *same* type of phrases (e.g., noun phrases). The multiple-branching constructions are made by conjoining phrases at the same hierarchical level, and the left/right-branching constructions are yielded by merging a phrase in the left-most or right-most phrase. The degrees of nesting and self-embedding have been already proposed to model the understanding of sentences [2]. By generalizing this attractive idea to include any constructions with merged phrases, we introduced DoM as a key computational concept.

We have proposed that various other "miscellaneous" operations that have been employed in the linguistics literature, such as Agree, Scope determination, Copy, etc., are in fact different manifestations of one and the same, i.e., more generalized, operation of Search [3]. Thus, Agree, which has been assumed to be an operation mainly responsible for the agreement of grammatical features, is actually not an operation specific to agreement, but rather is just an instance of the basic operation Search, when it assigns specific features. Human language, therefore, should minimally contain only two universal operations, Merge and Search.

As regards the formal symbol sequences beyond the bounds of finite state languages, three specific types of language have been discussed in the linguistic literature: (i) "counter language", (ii) "mirror-image language", and (iii) "copying language" [cf. [4], p. 21].

(i) *ab*, *aabb*, *aaabbb*, …, and in general, all sentences consisting of *n* occurrences of *a* followed by *n* occurrences of *b* and only these;
(ii) *aa*, *bb*, *abba*, *baab*, *aaaa*, *bbbb*, *aabbaa*, *abbbba*, …, and in general, all sentences consisting of a string *X* followed by the 'mirror image' of *X* (i.e., *X* in reverse), and only these;
(iii) *aa*, *bb*, *abab*, *baba*, *aaaa*, *bbbb*, *aabaab*, *abbabb*, …, and in general, all sentences consisting of a string *X* of *a*'s and *b*'s followed by the identical string *X*, and only these.

The counter language can be handled by a counting mechanism to match the number of each symbol, whereas the mirror-image language contains a mirror-image dependency, requiring more than a mere counter. If the number of symbols is not fixed (i.e., infinite), both of these languages are to be generated by context-free (simple) phrase structure grammars, while the copying language

with a cross-serial dependency clearly goes beyond the bounds of context-free phrase structure grammars, requiring a more powerful device, viz., context-sensitive phrase structure grammars or transformational grammars [5,6]. In the present study, the Reverse and Same under the string conditions took the above type (i) of $A^n B^n$. As regards the matching orders, the Reverse took the type (ii) of $A_2 A_1 B_1 B_2$ or $A_3 A_2 A_1 B_1 B_2 B_3$, while the Same took the type (iii) of $A_1 A_2 B_1 B_2$ or $A_1 A_2 A_3 B_1 B_2 B_3$.

Because the number of symbols is inevitably fixed (i.e., finite) in any actual experiments, it should be noted that any symbol sequences can be expressed by a regular (finite state) grammar, i.e., the least powerful grammar in the so-called Chomsky hierarchy. Therefore, one cannot claim in principle that individual grammars (e.g., context-free phrase structure vs. regular grammars) are differentially represented in the brain, and the neural representation of individual grammars was *not* within the scope of the present study. Besides various models examined, other nonstructural and nonsymbolic models with simple recurrent networks have been proposed to process some examples of even context-free and context-sensitive phrase structure languages, generalizing to some degree to longer strings than the training set [7]. However, these models do not account for any parametric modulation of activations in the present study, except the length of sentences.

Operational Definitions of All Factors Examined

We operationally defined syntactic factors within an entire sentence (see Table 1) as follows. If a tree structure [a Phrase-marker (P-marker) associated with a linguistic expression] contains as its subtree a domain in which a node *N* immediately dominates *n* elements ($n > 1$), then we can say that the domain constitutes a *merged* structure. Note that under the binary Merge hypothesis, *n* equals 2, except for relatively rare "multiple branching" structures (see Appendix S2). In the present study, we abstract away from the noun/verb (N/V or $\overline{N}/\overline{V}$) vs. noun phrase/verb phrase (NP/VP) distinction, as well as the sentence (S) vs. complementizer phrase (CP or $\overline{S}$) distinction. The operational definitions of syntactic factors examined here are as follows (see Figures 1A and 2A). "Number of Merge" is the total number of binary branches. "Number of Search" is the total number of correspondences between sample and comparison stimuli. DoM is the largest integer *m* meeting the following condition: there is a continuous path passing through $m + 1$ nodes $N_0, \ldots, N_m$, where each N_i ($i \geq 1$) is *merged* in the subtree dominated by N_{i-1}.

From both theoretical and experimental points of view, we also examined in detail the validity of other structure-based models, here categorized as "other linguistic factors". If a merged structure is surrounded by non-null elements on both sides, we get a "nested" structure. If a nesting structure occurs within the *same* type of elements, the structure is called a "self-embedded" structure. "Degree of nesting" (or "degree of self-embedding") is the largest integer *m* meeting the following condition (Figure S1A): there is a continuous path passing through $m + 1$ nodes $N_0, \ldots, N_m$, where each N_i ($i \geq 1$) is nested (or fully self-embedded) in the subtree dominated by N_{i-1} [2]. "Number of nodes" is the total number of nonterminal nodes and terminal nodes (Figure S1B).

As regards "depth of postponed symbols", its original definition was for *producing* a given output sequence [8]. As we tested stimuli for *understanding* a given input sequence, we reversed the numbering for the listener/reader as follows. "Depth of postponed symbols" is the amount of temporary storage needed for parsing a given input sequence, which can be calculated in the following way: first, number the branches of each node from 0 to $n - 1$ (Figure S2, the digits shown in red), where *n* is the number of branches from that node. Start numbering from the left. Then, compute the depth *d* of each terminal node by adding together the numbers written along all branches leading to that terminal node, starting from the leftmost branch (i.e., the first input for the listener/reader).

According to the dependency locality theory [9], two components of sentence parsing that consume computational resources have been proposed: "integration costs", which are connecting words into the structure for the input thus far, and "storage costs", which are the minimum number of words required to complete the current input as a grammatical sentence (Figure S3A). By adding

together “new discourse referents” (Ns and Vs in our paradigm) and “structural integrations” (the number of discourse referents in the intervening region), “integration costs” are obtained. According to the similarity-based interference theory [10], there are combined effects of “retroactive interference” (the number of nominative NPs between the subject-verb pair, when a verb is processed) and “proactive interference” (the number of nominative NPs, which are prior to the subject and still active in the parse) on syntactic attachments (Figure S3B). By adding both interference effects together, “syntactic interference” is obtained. Another source of interference is “positional similarity”, which is the number of adjacent syntactically similar NPs (i.e., marked with similar case markers).

Nonlinguistic factors may also variably contribute to the processing load of sentences with different constructions. At least three basic nonlinguistic factors may be involved in our experiment: memory span, counting, and “number of encoding”. The operational definitions of these nonlinguistic factors are as follows. Memory span is the maximum cost needed to maintain an item for matching against intervening or skipped stimuli (e.g., zero for N_1 V_1 in the $Conjoined_{(S)}$, and one for N_1 VV_1 in the $Simple_{(S)}$), and its operational definition is the maximum number of cusps in the curved arrows (Figures 1 and 2). Counting is an operation needed to track symbol repetition, and its operational definition is the maximum number of consecutively repeated symbols (e.g., zero for NVNV or NVNVNV, one for NNVV, and two for NNNVVV). Encoding is memorization of features necessary for matching, and “number of encoding” is the total number of sample and comparison stimuli. Memory span and counting were considered temporal order-related factors in our experiment; memory span was related to matching orders, while counting was related to symbol orders. On the other hand, memory span and “number of encoding” were memory-related factors.

References

1. Chomsky N (1965) Aspects of the Theory of Syntax. Cambridge, MA: The MIT Press. 251 p.
2. Miller GA, Chomsky N (1963) Finitary Models of Language Users. In: Luce RD, Bush RR, Galanter E, editors. Handbook of Mathematical Psychology. New York, NY: John Wiley and Sons. pp. 419-491.
3. Fukui N, Sakai H (2003) The visibility guideline for functional categories: Verb raising in Japanese and related issues. Lingua 113: 321-375.
4. Chomsky N (1957) Syntactic Structures. The Hague: Mouton Publishers. 117 p.
5. Chomsky N (1959) On certain formal properties of grammars. Inform Control 2: 137-167.
6. Hopcroft JE, Ullman JD (1979) Introduction to Automata Theory, Languages, and Computation. Reading, MA: Addison-Wesley. 418 p.
7. Rodriguez P (2001) Simple recurrent networks learn context-free and context-sensitive languages by counting. Neural Comput 13: 2093-2118.
8. Yngve VH (1960) A model and an hypothesis for language structure. Proc Am Philos Soc 104: 444-466.
9. Gibson E (2000) The Dependency Locality Theory: A Distance-Based Theory of Linguistic Complexity. In: Marantz A, Miyashita Y, O'Neil W, editors. Image, Language, Brain: Papers from the First Mind Articulation Project Symposium. Cambridge, MA: The MIT Press. pp. 95-126.
10. Lewis R, Nakayama M (2002) Syntactic and positional similarity effects in the processing of Japanese embeddings. In: Nakayama M, editor. Sentence Processing in East Asian Languages. Stanford, CA: CSLI Publications. pp. 85-111.
