# Supplementary material for: Syntactic Computation in the Human Brain: The Degree of Merger as a Key Factor
Source: PLoS One. 2013 Feb 20;8(2):e56230. doi: 10.1371/journal.pone.0056230 (PMC3577822; doi:10.1371/journal.pone.0056230)
Supplement: Appendix S2 — Detailed information about the stimuli. (PDF) [file pone.0056230.s008.pdf]

Syntactic Computation in the Human Brain: The Degree of Merger as a Key Factor

Shinri Ohta, Naoki Fukui, Kuniyoshi L. Sakai

Appendix S2 Detailed information about the stimuli

In the Japanese language, all regular verbs are either of *ichidan* (one-tier) or *godan* (five-tier) conjugation [1]. In our experiments, Vs took a nonpast-tense form (*-ru*), past-tense form (*-ta*), or gerundive form (*-te*) (e.g., "*teteru*", "*teteta*", and "*tetete*"), inflecting like normal *ichidan* verbs (e.g., "*tateru*", "*tateta*", and "*tatete*": "*build*", "*built*", and "*building*"). In a subordinate clause, Vs took tense markers (*-ru*/*-ta*) just before *-to*. The last V in an entire sentence always ended with tense markers. As the tense of verbs can be independently marked in a Japanese nested sentence (e.g., "*Taro-ga Hanako-ga utau-to omotta*": "*Taro thought that Hanako would sing*"), we regarded "number of tense markers (*-ru*/*-ta*)" as one of other linguistic factors (Table 1).

In Japanese, there are a number of morphologically/semantically related pairs of transitive and intransitive verbs with vowel changes (e.g., "*kakeru*" and "*kakaru*": "*hang up*" and "*hang down*") [2]. Not only such vowel dissimilation, but vowel assimilation, i.e., *vowel harmony*, is commonly observed in natural languages [3]. Vowel harmony is possible between adjacent or distant vowels within a word [4]. Indeed, the presence of vowel harmony has been indicated in the history of the Japanese language [1]. In the present study, vowel harmony was adopted to change the last, i.e., second, vowel of the verb root, so that this vowel harmonized with the vowel (i.e., /a/, /u/, or /o/) of the corresponding subject (e.g., "*rara-ga tetaru*" and "*rara-ga tetatta*" from "*teteru*" and "*teteta*", respectively). This property of vowel harmony made each V_i inflect like a normal *godan* verb, which root always ends in a consonant, and this change made V_i distinct from the original form of *ichidan* verbs. It should be noted that V_i of nonmatching stimuli also inflected like normal *godan* verbs (Tables S1 and S2). When a *godan* verb root ends in *r*, *t*, or *w*, an euphonic change of a geminated consonant *t* occurs before *-ta* or *-te* (e.g., "*kakatta*" from "*kakari-ta*": "*hung down*"). Each V_i with *-ta* or *-te* (e.g., "*tetatta*") was consistent with its adverbial form *ri* ("*tetari-ta*"), *ti* ("*tetati-ta*"), or (*w*)*i* ("*tetai-ta*"). On the other hand, the first verb of a compound verb remained an *ichidan* verb in an adverbial form (e.g., "*tete*").

There are some grammatical, but noncanonical (i.e., in a special use), usages of *-ga* or *-no*: parallel subjects marked with *-ga* (e.g., "*Taro-ga yuujin-ga sorezore utatta*": "*Taro and his friend each sang*"), an object marked with *-ga* (e.g., "*Taro-ga yuujin-ga suki-da*": "*Taro likes his friend*"), an external possessor marked with *-ga* (e.g., "*Taro-ga yuujin-ga sinsetu-da*": "*Taro's friend is kind*"), and a subject marked with *-no* (e.g., "*Taro-no suki-na yuujin*": "*the friend Taro likes*"). Considering such canonicity, we regarded "number of case markers (*-ga*/*-no*)" as one of other linguistic factors (Table 1). However, these noncanonical case markings are rare in both comprehension and production, as shown by previous behavioral experiments [5,6]. We assured the participants that case markings for our stimuli were always canonical (see Appendix S3). Actual usage of canonical case markings was fully guaranteed by the high accuracy under the sentence conditions (Figure 3A), as the matching task could not be performed correctly if such noncanonical case markings were employed.

We imposed three following constraints for the letter strings. First, for the first half of a string (As), letter strings derived from Ns and Vs (denoted here as $\mathcal{N}$s and $\mathcal{V}$s, respectively) were in the order of $\mathcal{N}\mathcal{V}$ or $\mathcal{V}\mathcal{N}$ for the short stimuli, and $\mathcal{N}\mathcal{V}\mathcal{N}$ or $\mathcal{V}\mathcal{N}\mathcal{V}$ for the long stimuli. Secondly, we avoided endings with *-ga*/*-no* for $\mathcal{N}$s, but some $\mathcal{V}$s with *-ru*/*-ta* endings were used as stimuli. Lastly, neither Vs with *-to*/*-te* endings nor Vs in the adverbial form were used for making $\mathcal{V}$s.

Examples of long sentences are shown in Figure 2A. For the $\text{Nested}_{(L)}$, a sentence at the lowest hierarchical level (S) was self-embedded twice into an entire sentence (S"). For the $\text{Simple}_{(L)}$, we tested both stimuli of $(VV_1)V_1$ and $V_1(VV_1)$, where two verbs, i.e., VV_1 (a compound verb) and V_1, were conjoined. As regards the Ns in the $\text{Simple}_{(L)}$, branching constructions were ambiguous between $(NN)N_1$ and $N(NN_1)$, like "*Japanese history teacher*" ([[*Japanese history*] *teacher*] and [*Japanese* [*history teacher*]], respectively), both of which yielded the same DoM. For Japanese relative clauses, as well as for noun phrases with a genitive case marker *-no*, left-branching constructions predominate [cf. [7], pp.471-472]. For the $\text{Conjoined}_{(L)}$, DoM for a node was increased by one from the highest nodes of an entire sentence (S') to the same hierarchical level of conjoined sentences (Ss). Since all the conjoined sentences were equivalent with respect to their status in the multiple branching, two Merge applications involved here were assumed to follow, as a marked (i.e., "exceptional") option, the associative law (i.e., $[[a \# b] \# c] = [a \# [b \# c]]$, where # represents Merge). This type of associative Merge (which yields *n*-ary structures by applying $n - 1$ times) has been argued to be permissible for a certain class of marked constructions in human language [8]. In this way, the number of Merge in a sentence becomes always one less than the number of terminal nodes, *irrespective of sentence structures*.

References

1. Shibatani M (1990) The Languages of Japan. Cambridge, UK: Cambridge University Press. 411 p.
2. Tsujimura N (2007) An Introduction to Japanese Linguistics, 2nd Edition. Malden, MA: Blackwell Publishing. 501 p.
3. Nevins A (2010) Locality in Vowel Harmony. Cambridge, MA: The MIT Press. 244 p.
4. Mailhot F, Reiss C (2007) Computing long-distance dependencies in vowel harmony. Biolinguistics 1: 28-48.
5. Uehara K, Bradley DC (2002) Center-embedding Problem and the Contribution of Nominative Case Repetition. In: Nakayama M, editor. Sentence Processing in East Asian Languages. Stanford, CA: CSLI Publications. pp. 257-287.
6. Miyamoto ET (2002) Case markers as clause boundary inducers in Japanese. J Psycholinguist Res 31: 307-347.
7. Miller GA, Chomsky N (1963) Finitary Models of Language Users. In: Luce RD, Bush RR, Galanter E, editors. Handbook of Mathematical Psychology. New York, NY: John Wiley and Sons. pp. 419-491.
8. Fukui N (2011) Merge and Bare Phrase Structure. In: Boeckx C, editor. The Oxford Handbook of Linguistic Minimalism. Oxford, UK: Oxford University Press. pp. 73-95.
