# Supplementary material for: Syntactic Computation in the Human Brain: The Degree of Merger as a Key Factor
Source: PLoS One. 2013 Feb 20;8(2):e56230. doi: 10.1371/journal.pone.0056230 (PMC3577822; doi:10.1371/journal.pone.0056230)
Supplement: Appendix S3 — Task instructions and training procedures. (PDF) [file pone.0056230.s009.pdf]

Syntactic Computation in the Human Brain: The Degree of Merger as a Key Factor

Shinri Ohta, Naoki Fukui, Kuniyoshi L. Sakai

Appendix S3 Task instructions and training procedures

Task Instructions

Before the experiments, all participants were fully informed about the stimuli and task. We instructed and trained the sentence conditions in the order of $\text{Simple}_{(S)}$, $\text{Conjoined}_{(S)}$, $\text{Nested}_{(S)}$, $\text{Simple}_{(L)}$, $\text{Conjoined}_{(L)}$, and $\text{Nested}_{(L)}$, as the number of vowel extraction increased in this order. The following is a translation of task instructions in Japanese.

[*Day 1 instructions*]

Words used in a task

Special pseudowords and letter strings will be used in a task. ***You don't have to remember the following words***.

Pseudowords used as nouns (six kinds) are: "*rara*", "*zaza*", "*mumu*", "*gugu*", "*yoyo*", and "*dodo*".

Grammatical particles attached to the noun (two kinds) are: "*-ga*", which marks a subject, and "*-no*", which marks a modifier (e.g., "*zaza-ga*", "*mumu-no*", etc.).

Pseudowords used as verbs (four kinds) are: "*hihi*", "*kiki*", "*sese*", and "*tete*".

Verb endings (five kinds) and verb conjugation patterns are:

1. Verb endings: "*-ru*" (present tense), "*-tta*" (past tense), "*-tte*" (e.g., "*utatte*"), "*-ru-to*" (e.g., "*odoru-to*"), and "*-tta-to*" (e.g., "*odotta-to*"); and
2. Verb conjugation: ***When a subject corresponds to a verb within a sentence****, the latter vowel* (second syllable) *of the verb root will change in order to match the vowel of the corresponding subject.* You don't have to recall the root form of the verb.

Correct examples:

"*rara-ga hiharu*" (conjugation of "*hihi*")
"*mumu-ga kikutta*" (conjugation of "*kiki*")
"*dodo-ga sesoru*" (conjugation of "*sese*")
"*rara-ga tetatta*" (conjugation of "*tete*")

Wrong examples:

"*zaza-ga hihoru*"
"*gugu-ga kikotta*"
"*yoyo-ga sesuru*"
"*zaza-ga tetoru*"

Other letter strings are: "*ragara*", "*nogugu*", "*huhhita*", "*kottaki*", etc.

Types of conditions

1) Grammar (G) conditions, 2) Memory (M) condition, and 3) Reverse (R) condition.

You will perform a task under three types of conditions during scanning of your head images inside the MRI scanner. At the beginning of each trial, a cue ("G", "M", or "R") denoting a condition will appear. By noting these cues, please judge which condition will be presented. During the task, a small red cross remains at the center of the monitor. Fixate this red cross as much as you can, but you may blink. *Please do not speak or read aloud during the task.*

During the task, please respond by pressing a button. Hold a switch box by both hands, with a red button to your right. When you press one, use your right thumb without looking at the buttons. Please note the following crucial points:

- Press a button only once, when necessary.
- *Please press the button as fast as you can, while the stimuli are presented.*
- Every stimulus is consisted of a cue denoting a condition and of four or six phrases or strings.
- *Do not press a button before the last stimulus*.

1. Grammar conditions

There are six patterns under the Grammar conditions. The task is to judge whether or not the vowel of a subject is matched with the last vowel of the corresponding verb root. At the beginning of each trial, the cue "G" denoting a condition will appear. Do not overlook this cue, so that you can answer correctly. For every pattern shown below, "G" will appear.

1.1 Pattern 1 (Simple$_{(S)}$)

Pattern 1 is similar to "*Taro-no ani-ga sinobi aruita*" ("*Taro's brother sneaked around*"). The subject is "*ani*", and the verb is "*aruita*". Please judge whether or not the vowel of the *second* phrase (subject) is matched with that of the *fourth* phrase (corresponding verb). Please press *the rightmost button* if correct, and press *the second one from the right* if wrong. Press the button quickly with your right thumb, while the fourth stimulus is presented.

Correct examples:
"*zaza-no yoyo-ga hihi tetoru*"
"*zaza-no yoyo-ga hihi tetotta*"

Wrong examples:
"*zaza-no yoyo-ga tete sesuru*"
"*zaza-no yoyo-ga tete sesatta*"

1.2 Pattern 2 (Conjoined$_{(S)}$)

Pattern 2 is similar to "*Taro-ga utatte Hanako-ga odoru*" ("*Taro sings, and Hanako dances*"). Please judge whether or not the vowel of the *first* phrase is matched with that of the *second* phrase, and the vowel of the *third* phrase with that of the *fourth* phrase.

Correct examples:
"*zaza-ga tetatte yoyo-ga kikoru*"
"*zaza-ga tetatte yoyo-ga kikotta*"

Wrong examples:
"*zaza-ga tetatte yoyo-ga kikuru*"
"*zaza-ga tetotte yoyo-ga kikatta*"

1.3 Pattern 3 (Nested$_{(S)}$)

Pattern 3 is similar to "*Taro-ga Hanako-ga utau-to omotta*" ("*Taro thought that Hanako would sing*"). Please judge whether or not the vowel of the *first* phrase is matched with that of the *fourth* phrase, and the vowel of the *second* phrase with that of the *third* phrase.

Correct examples:
"*zaza-ga yoyo-ga tetoru-to kikaru*"
"*zaza-ga yoyo-ga tetotta-to kikatta*"

Wrong examples:
"*zaza-ga yoyo-ga tetoru-to kikoru*"
"*zaza-ga yoyo-ga tetatta-to kikutta*"

Tips

1. Please do not forget the first noun.
2. There are sentences that have multiple errors; *please check them up to the last phrase*.
3. Please press the button *as fast as you can, while the last phrase is presented*.
4. *When the last phrase disappears, please quit the button press, and concentrate on the next trial*.

2. Memory condition (Same$_{(S)}$)

The task is to memorize the presented letter strings. At the beginning of each trial, the cue "M" denoting a condition will appear. Do not overlook this cue, so that you can answer correctly.

Four strings will appear one by one on the monitor. Please memorize these four strings, and judge whether or not the *first* and *third* strings, and the *second* and *fourth* ones, are exactly the same.

Correct examples:

"*yogayo tarute yogayo tarute*"
"*hiruhu garara hiruhu garara*"

Wrong examples:

"*yonoyo tettata yonoyo tatetta*"
"*hihhata nogugu settaso gunogu*"

3. Reverse condition (Reverse$_{(S)}$)

The task is to memorize the presented letter strings. At the beginning of each trial, the cue "R" denoting a condition will appear. Do not overlook this cue, so that you can answer correctly.

Four strings will appear one by one on the monitor. Please memorize these four strings, and judge whether or not the *first* and *fourth* strings, and the *second* and *third* ones, are exactly the same.

Correct examples:

"*yogayo tarute tarute yogayo*"
"*hiruhu garara garara hiruhu*"

Wrong examples:

"*yonoyo tettata tatetta yonoyo*"
"*hihhata nogugu nogugu settaso*"

[*Day 2 instructions*]

1. Grammar conditions

1.1 Pattern 4 (Simple$_{(L)}$)

Pattern 4 is similar to "*Taro-no ani-no yujin-ga hasiri mawatte utatta*" ("*Taro's brother's friend run around and sang*"). The subject is "*yujin*", and the verbs are "*mawatte*" and "*utatta*". Please judge whether or not the vowel of the *third* phrase is matched with that of the *fourth* or *fifth* phrase, and the vowel of the *third* phrase with that of the *sixth* phrase. Please press *the rightmost button* if correct, and press *the second one from the right* if wrong. Press the button quickly with your right thumb, while the sixth stimulus is presented.

Correct examples:

"*zaza-no yoyo-no mumu-ga tete kikutte hihuru*"

"*zaza-no yoyo-no mumu-ga tetutte kiki hihutta*"

Wrong examples:

×
"*zaza-no yoyo-no mumu-ga tetutte kiki hiharu*"

×
×
"*zaza-no yoyo-no mumu-ga tete kikatte hihotta*"

1.2 Pattern 5 (Conjoined$_{(L)}$)

Pattern 5 is similar to "*Taro-ga odotte Hanako-ga utatte Jiro-ga asobu*" ("*Taro dances, Hanako sings, and Jiro plays*"). Please judge whether or not the vowel of the *first* phrase is matched with that of the *second* phrase, the vowel of the *third* phrase with that of the *fourth* phrase, and the vowel of the *fifth* phrase with that of the *sixth* phrase.

Correct examples:

"*zaza-ga tetatte yoyo-ga kikotte mumu-ga hihuru*"

"*zaza-ga tetatte yoyo-ga kikotte mumu-ga hihutta*"

Wrong examples:

"*zaza-ga tetatte yoyo-ga kikotte mumu-ga hiharu*"

"*zaza-ga tetutte yoyo-ga kikotte mumu-ga hihatta*"

1.3 Pattern 6 (Nested$_{(L)}$)

Pattern 6 is similar to "*Taro-ga Hanako-ga Jiro-ga utau-to omou-to kangaeta*" ("*Taro supposed that Hanako would think that Jiro would sing*"). Please judge whether or not the vowel of the *first* phrase is matched with that of the *sixth* phrase, the vowel of the *second* phrase with that of the *fifth* phrase, and the vowel of the *third* phrase with that of the *fourth* phrase.

Correct examples:

"*zaza-ga yoyo-ga mumu-ga teturu-to kikoru-to hiharu*"

"*zaza-ga yoyo-ga mumu-ga tetutta-to kikotta-to hihatta*"

Wrong examples:

"*zaza-ga yoyo-ga mumu-ga teturu-to kikoru-to hihoru*"

"*zaza-ga yoyo-ga mumu-ga tetutta-to kikatta-to hihotta*"

2. Memory condition (Same$_{(L)}$)

Six strings will appear one by one on the monitor. Please memorize these six strings, and judge whether or not the *first* and *fourth* strings, the *second* and *fifth* ones, and the *third* and *sixth* ones, are exactly the same.

Correct examples:

"*yogayo teruta gagugu yogayo teruta gagugu*"
"*huruhi garara ruseso huruhi garara ruseso*"

Wrong examples:

"*noyoyo tattate gunogu noyoyo tattate munomu*"
"*huttahi ranora sottase hihhuta ranora tattate*"

3. Reverse condition (Reverse$_{(L)}$)

Six strings will appear one by one on the monitor. Please memorize these six strings, and judge whether or not the *first* and *sixth* strings, the *second* and *fifth* ones, and the *third* and *fourth* ones, are exactly the same.

Correct examples:

"*yogayo teruta gagugu gagugu teruta yogayo*"
"*huruhi garara ruseso ruseso garara huruhi*"

Wrong examples:

"*noyoyo tattate gunogu munomu tattate noyoyo*"
"*huttahi ranora sottase tattate ranora hihhuta*"

Training Procedures

Experiments with short or long stimuli were performed on separate days in the order short, then long. Before scanning, the participants were trained until they scored 80 % at each of the following stages. Each of the Simple, Conjoined, and Nested were separately tested with self-paced reading in 10 to 30 trials with this order. Only one participant took 50 trials for the Nested$_{(L)}$. When

these conditions were randomized, all participants took additional 20 or 40 trials. The Reverse and Same were much easier to perform, and so required only 10 or 20 trials for a randomized sequence from the beginning. When all of these conditions were mixed, participants additionally took 20 or 40 trials. Finally, participants were tested on a sequence of all conditions at the regular rate of presentation (see Figure 1C and 1D) in 40 and 20 trials for the short and long stimuli, respectively. Only three participants required 60 or 80 trials for the short stimuli. Due to technical problems, four participants were retested with short or long stimuli on another day; two participants received 20 trials for the mixed conditions with self-paced reading, and all received 20–60 trials for the final stage.
