# Supplementary material for: Syntactic Computation in the Human Brain: The Degree of Merger as a Key Factor
Source: PLoS One. 2013 Feb 20;8(2):e56230. doi: 10.1371/journal.pone.0056230 (PMC3577822; doi:10.1371/journal.pone.0056230)
Supplement: Appendix S4 — MRI data analyses. (PDF) [file pone.0056230.s010.pdf]

Syntactic Computation in the Human Brain: The Degree of Merger as a Key Factor

Shinri Ohta, Naoki Fukui, Kuniyoshi L. Sakai

Appendix S4 MRI data analyses

fMRI Data Analyses

Data analyses with fMRI were performed using SPM5 statistical parametric mapping software (Wellcome Department of Cognitive Neurology, London, UK; http://www.fil.ion.ucl.ac.uk/spm/) [1] on MATLAB software (Math Works, Natick, MA). The acquisition timing of each slice was corrected using the middle slice as a reference for the EPI data. We realigned the EPI data in multiple runs to the first volume in multiple runs, and removed runs that included data with a translation of > 2 mm in one of the three directions and with a rotation of > 1.4° around one of the three axes. Each participant's T1-weighted image was coregistered to the mean image of its own EPI data, followed by the segmentation of the T1-weighted image into grey- and white-matter probability maps. With this latter step, each individual brain was spatially normalized to the standard brain space as defined by Montreal Neurological Institute (MNI). Resampling was performed every 3 mm using seventh-degree B-spline interpolation, and images were smoothed with an isotropic Gaussian kernel of 9 mm full width at half maximum (FWHM).

Using these preprocessed data, low-frequency noise and global changes in activity were further removed (cut-off period: 128 s). Hemodynamic responses induced by the trials were modeled with a box-car function with a duration of 3.5 s (short stimuli) or 5.1 s (long stimuli), i.e., from the onset of the first stimulus (N or A) to 500 ms after the disappearance of the last stimulus (Figure 1C and 1D), and the box-car function was convolved with a hemodynamic function that peaked at 6 s. These functions were used as run-specific covariates for matching or nonmatching stimuli for each condition in a general linear model (GLM). Only event-related responses to correct trials were analyzed.

For random effects analyses using either an analysis of covariance (ANCOVA) with *t*-statistics or a one-sample *t*-test, contrast images were generated for each participant and used for intersubject comparisons. For all fMRI data analyses, the statistical threshold was set to $P < 0.05$ for the voxel level, corrected for multiple comparisons [family-wise error (FWE) correction] across the whole brain. To exclude any general cognitive factors related to task difficulty from the evaluation of cortical activation, the accuracy was used as a nuisance variable for each contrast of random effects analyses. If a subtraction between conditions served as a reference, an exclusive mask (uncorrected $P < 0.01$) was applied to reduce the contribution of deactivation. For example, in Nested' > Simple', an exclusive mask of the [Conjoined – Simple] contrast was applied to reduce the contribution of deactivation in Simple'.

We used a factorial design for a two-way ANCOVA with condition [Nested', Simple'] × length [Long, Short], where activations in Nested' or Simple' for long or short sentences were estimated for each participant, and then the main contrast (as denoted by a greater-than symbol) was calculated for intersubject comparisons. A one-way ANCOVA was used for a direct comparison of Nested'$_{(L)}$ > Simple'$_{(S)}$, and for Nested" > Reverse" contrasting the sentence and string conditions. To ensure an independent statistical test in the latter contrast, the inclusive mask of Nested' > Simple' (corrected $P < 0.05$) was iteratively applied to the contrast image of each participant, thereby leaving out that participant from the calculation of the mask, i.e., the "leave-one-subject-out cross-validation" approach [2]. One-sample *t*-test was used for [Nonmatching – Matching] separately under the sentence and string conditions, and for Reverse".

For the anatomical identification of activated regions, we basically used the Anatomical Automatic Labeling method [3]. The percent signal changes averaged across the voxels in each activated region were extracted using the MarsBaR-toolbox (http://marsbar.sourceforge.net/). To fit a model of each factor to activations, a fitting scale and residual sum of squares (RSS) were calculated with MATLAB, and the fitted values were obtained by multiplying the estimates by the fitting scale. For a no-intercept model, coefficient of determination (r^2) should be calculated as $r^2 = 1 - \Sigma(y - \hat{y})^2 / \Sigma y^2$, where $\hat{y}$ and y denote the fitted values and the signal changes for each contrast, respectively [4]. For this calculation, we used R software (http://www.r-project.org/). By using a restricted maximum-likelihood method, we further fitted "linear mixed-effects models" with individual activations as dependent variables, the estimates of each factor as a regressor, and the participants as random effects. For this calculation, we used an nlme (linear and nonlinear mixed-effects models) package (http://cran.r-project.org/web/packages/nlme/) on R software.

DCM Data Analyses

Data analyses with DCM were performed using DCM10 on SPM 8 [5]. For DCM analyses, we concatenated the scans from the separate runs, and reanalyzed the preprocessed data with GLM, which contained regressors representing the Nested, Simple, Reverse, and Same (correct trials alone; see Table 2), as well as a regressor representing all conditions (correct trials alone including Conjoined). In addition, the effects of transition between runs were taken into account by adding regressors for each run.

We imposed following functional and anatomical constraints for selecting ROIs (regions of interest) in L. F3op/F3t or L. SMG for each participant with the individually preprocessed data [6]. The individual local maxima should be significant in Nested" – Reverse" estimated for each participant (uncorrected $P < 0.05$), nearest to the group local maxima of Nested" > Reverse" within twice the FWHM of the smoothing kernel, and obviously in L. F3op/F3t or L. SMG. The averaged MNI coordinates of these individual local maxima were (–50, 26, 25) and (–39, –45, 43) for L. F3op/F3t and L. SMG, respectively. With the volume-of-interest (VOI) tool on SPM8, the time series was extracted by taking the first eigenvariate across all suprathreshold voxels within 6 mm of the individual local maxima.

We specified nine models with systematic variations in a modulatory effect and driving inputs (Figure S4). The regressor representing the Nested was used for a modulatory effect, whereas that representing all conditions was used for driving inputs. After estimating all models for each participant, we identified the most likely model by using random-effects Bayesian model selection (BMS) on DCM10. Inferences from BMS can be based on the expected probability, i.e., the expected likelihood of obtaining the model for any randomly selected participants, or on the exceedance probability, i.e., the probability that the model is a better fit to the data than any other models tested. We adopted the family inference method [7], in which three modulatory families were compared first, and then the input models within the winning family were further compared. After determining the best model, the parameter estimates of this particular model were evaluated by a one-sample *t*-test [8].

DTI Data Analyses

Data analyses with DTI were performed using FSL [Oxford Centre for Functional MRI of the Brain's (FMRIB) Software Library 4.1.7; http://fsl.fmrib.ox.ac.uk/fsl/fslwiki/] and FDT (FMRIB's Diffusion Toolbox 2.0) [9]. Diffusion-weighted images were first resliced to isotropic voxel of 1 mm^3, and then eddy current distortions and motion artifacts were corrected using affine registration to the b0 image. We then extracted the brain shape from the b0 image, and created the binary mask image (i.e., zero for the outside of the brain) for each participant. Markov Chain Monte Carlo sampling was performed to build up distributions on diffusion parameters at each voxel, which allowed for an estimation of the most probable pathway by Bayesian estimation (number of fibers

modeled per voxel = 2) [10]. The implicit modeling of noise in a probabilistic model made a fiber tracking near grey matter possible.

By using FLIRT (FMRIB's Linear Image Registration Tool) on FSL, the b0 image was first coregistered to the individual T1-weighted image for each participant, and the T1-weighted image was spatially normalized to the MNI space by using both affine and nonlinear transformations with FLIRT and FNIRT (FMRIB's Nonlinear Image Registration Tool). With the transformation matrices and estimated deformation fields, the peak MNI coordinates of each local maximum were transformed to the individual b0 images, and a sphere of 6-mm radius centered at the new coordinates was defined as a seed mask for the probabilistic tractography. All fiber tracking was conducted in an individual DTI space. In order to find the connections between these regions, we employed a two-ROI approach with two seed masks, which repeatedly sampled tracts from one seed mask, and retained only those tracts that passed through the other seed mask. Probabilistic fiber tracking was initiated from all voxels within the seed masks to generate 10,000 streamline samples, with a step length of 0.5 mm, a maximum number of steps of 2,000, a curvature threshold of 0.2 (±78.5°), and a loopcheck option.

In the connectivity distributions obtained, each voxel value represented the total number of the streamline samples passing through that voxel. The connectivity probability maps were then created for each participant by dividing the connectivity distributions with a sum of waytotal values, i.e., the total number of generated tracts from one seed mask that have reached the other seed mask. This normalization approach allowed for a comparison of connectivity probability values across participants; note that the pattern of connectivity did not change by this scaling. To remove any spurious connections, pathways in individual participants were thresholded to include only voxels that had at least 1 % connectivity probability values [11]. Thresholded pathways in each participant were then normalized as above and binarized using "fslmaths" on FSL. The binarized pathways were overlaid across participants to produce population probability maps for each pathway, in which voxel values represent the number of participants with a pathway through that voxel. These population probability maps with thresholding (at least seven out of 15 participants) were smoothed and shown using MRIcroN software (http://www.mccauslandcenter.sc.edu/mricro/mricron/).

References

1. Friston KJ, Holmes AP, Worsley KJ, Poline J-P, Frith CD et al. (1995) Statistical parametric maps in functional imaging: A general linear approach. Hum Brain Mapp 2: 189-210.
2. Esterman M, Tamber-Rosenau BJ, Chiu Y-C, Yantis S (2010) Avoiding non-independence in fMRI data analysis: Leave one subject out. Neuroimage 50: 572-576.
3. Tzourio-Mazoyer N, Landeau B, Papathanassiou D, Crivello F, Etard O et al. (2002) Automated anatomical labeling of activations in SPM using a macroscopic anatomical parcellation of the MNI MRI single-subject brain. Neuroimage 15: 273-289.
4. Kvålseth TO (1985) Cautionary note about R^2. Am Stat 39: 279-285.
5. Friston KJ, Harrison L, Penny W (2003) Dynamic causal modelling. Neuroimage 19: 1273-1302.
6. Stephan KE, Marshall JC, Penny WD, Friston KJ, Fink GR (2007) Interhemispheric integration of visual processing during task-driven lateralization. J Neurosci 27: 3512-3522.
7. Penny WD, Stephan KE, Daunizeau J, Rosa MJ, Friston KJ et al. (2010) Comparing families of dynamic causal models. PLoS Comput Biol 6, e1000709: 1-14.
8. Stephan KE, Penny WD, Moran RJ, den Ouden HEM, Daunizeau J et al. (2010) Ten simple rules for dynamic causal modeling. Neuroimage 49: 3099-3109.
9. Smith SM, Jenkinson M, Woolrich MW, Beckmann CF, Behrens TEJ et al. (2004) Advances in functional and structural MR image analysis and implementation as FSL. Neuroimage 23: S208-S219.

10. Behrens TEJ, Berg HJ, Jbabdi S, Rushworth MFS, Woolrich MW (2007) Probabilistic diffusion tractography with multiple fibre orientations: What can we gain? Neuroimage 34: 144-155.
11. Flöel A, de Vries MH, Scholz J, Breitenstein C, Johansen-Berg H (2009) White matter integrity in the vicinity of Broca's area predicts grammar learning success. Neuroimage 47: 1974-1981.
